# Grey Partridge (*Perdix perdix*) Introductions: Genetic Survey on Wild and Captive Populations at the Edges of the Range

**DOI:** 10.1002/ece3.71122

**Published:** 2025-03-21

**Authors:** N. Karaiskou, Α. Ball, K. Gkagkavouzis, A. Moulistanos, K. Kalaentzis, M. Ghazali, G. Murray‐Dickson, S. Minoudi, D. Parish, E. Chatzinikos, D. Kiousis, N. Manios, J. C. Dunn, A. Triantafyllidis

**Affiliations:** ^1^ Department of Genetics, Development & Molecular Biology, School of Biology, Faculty of Science Aristotle University of Thessaloniki Thessaloniki Greece; ^2^ Genomics and Epigenomics Translational Research (GENeTres), Center for Interdisciplinary Research and Innovation (CIRI‐AUTH) Balkan Center Thessaloniki Greece; ^3^ RZSS WildGenes Laboratory Royal Zoological Society of Scotland Edinburgh UK; ^4^ Hydrobiological Station of Rhodes Hellenic Centre for Marine Research Rhodes Greece; ^5^ Game & Wildlife Conservation Trust Fordingbridge Hampshire UK; ^6^ 4th Hunting Federation of Sterea Hellas Athens Greece; ^7^ Ministry of Environment and Energy Athens Greece; ^8^ School of Life Sciences Keele University Newcastle‐Under‐Lyme Staffordshire UK; ^9^ School of Biology University of Leeds Leeds UK

**Keywords:** grey partridge, introduction practices, phylogeography, population structure

## Abstract

The grey partridge population has experienced significant declines across Europe, largely due to agricultural intensification and loss of habitat, leading to conservation actions such as Red‐listing in the UK and hunting bans in Greece. The genetics of Balkan and Scottish populations remain largely unexplored; genetic analyses are essential to evaluate the impact of past introduction efforts on wild populations, as breeding between released and wild‐living partridges may complicate recovery efforts. In this study, we sample wild and farmed individuals of grey partridge from the Balkans (Greece, North Macedonia) and the United Kingdom (UK) and employ 2300 SNPs, eight microsatellites, and two mitochondrial markers to investigate the genetic structure and diversity of their populations and the impact of past release activities. We reveal a clear distinction between two clades, an Eastern and a Western, as in previous studies, with wild birds from Greece and the UK classified to each clade, respectively. However, birds from North Macedonia belonged to either clade, suggesting a contact zone between the two or a genetic legacy of past introduction practices. The captive stock in Greece and the UK is clearly of Western origin, with minor introgression of the Eastern clade being detected. Finally, an informative SNP marker panel is presented that accurately assigns each individual to either the Eastern or Western clade and will serve as a valuable tool for monitoring population structure, guiding conservation efforts, and assessing the impact of introduction events on grey partridge populations.

## 1 Introduction

When undertaking any reintroduction program, it is important to consider the geographic origin of the released individuals and the original population. Introgression between nonlocal translocated organisms and local wild populations can have unforeseen genetic effects on the indigenous population since it potentially affects local adaptation in the native wild population when pairing between translocated and native individuals takes place (Randi 2008).

Although the grey partridge ( *Perdix perdix* ) is considered to be common and not threatened on a global scale, its populations appear to be declining in intensively farmed areas, most likely due to a reduction in breeding sites and food stocks (Ewald et al. 2020). In Europe, since the beginning of the 20th century, there has been a drastic decline in grey partridge populations in all of the 31 countries where seven recognized subspecies exist (Kuijper et al. 2009). For example, its breeding abundance fell by 92% from 1967 to 2022 across the UK, probably due to the intensification of agriculture (BirdTrends 2023, www.bto.org/birdtrends). For this reason, the grey partridge has been Red‐listed as a Bird of Conservation Concern, and the British government launched a major programme to help partridge recovery in the UK nearly 20 years ago (Aebischer and Ewald 2012). Population shrinkage is also observed in southern populations of grey partridge, like Greece, where, mainly from 1950 and onward, there has been a drastic population decline (Thomaides and Papageorgiou 1992). For this reason, the hunting of grey partridge has been prohibited in Greece since 1984. Releases in Greece have also taken place with farmed individuals imported mainly from countries in the Balkans and Northern Europe of unknown provenance. Several studies have clearly shown that the survival of hand‐reared birds is poor after their release into the wild (Liukkonen 2006; Parish and Sotherton 2007). It has been suggested that differences in the genetic adaptation of separate subspecies (*P. p. lucida*, *P. p. perdix*) to different climatic conditions of their original range could be a partial explanation for the failed introductions (Liukkonen 2006). Therefore, it is of utmost importance that a genetic analysis of wild populations is carried out.

Genetic provenance of individuals is a key assessment criterion in the IUCN guidelines for reintroductions and translocations (IUCN 2013) as both inbreeding and outbreeding depression are potential concerns. Some genetic information is available for European populations of grey partridge; phylogeographical analysis of 227 birds from several localities based on mitochondrial DNA sequencing revealed a distinction of populations in two major clades (western and eastern) in agreement with the subspecies taxonomy (Liukkonen 2006). However, little is known about the genetics of range‐edge populations, such as those in Scotland and the Southern Balkans, Greece, and North Macedonia. No individuals have been sampled so far from Scotland, while two genetic studies with < 10 individuals from Greece using mtDNA and microsatellite data respectively revealed that the Greek population belongs to the eastern group (Liukkonen‐Anttila et al. 2002; Liukkonen et al. 2012).

We use three different molecular markers (mtDNA, microsatellite and SNP) to study a Northern and a Southern wild and farmed European population of grey partridge, comparing the provenance of each. The aim was to (1) shed light on the genetic structure and the genetic diversity of Balkan and UK wild populations, and (2) investigate the introgressive impact of past introduction events on the genetic pool of wild populations. Our results could have implications for the sourcing of released birds, since the breeding of released stock with wild partridges may complicate the recovery of partridge populations.

## 2 Materials and Methods

### 2.1 Sample Collection

**FIGURE 1:**
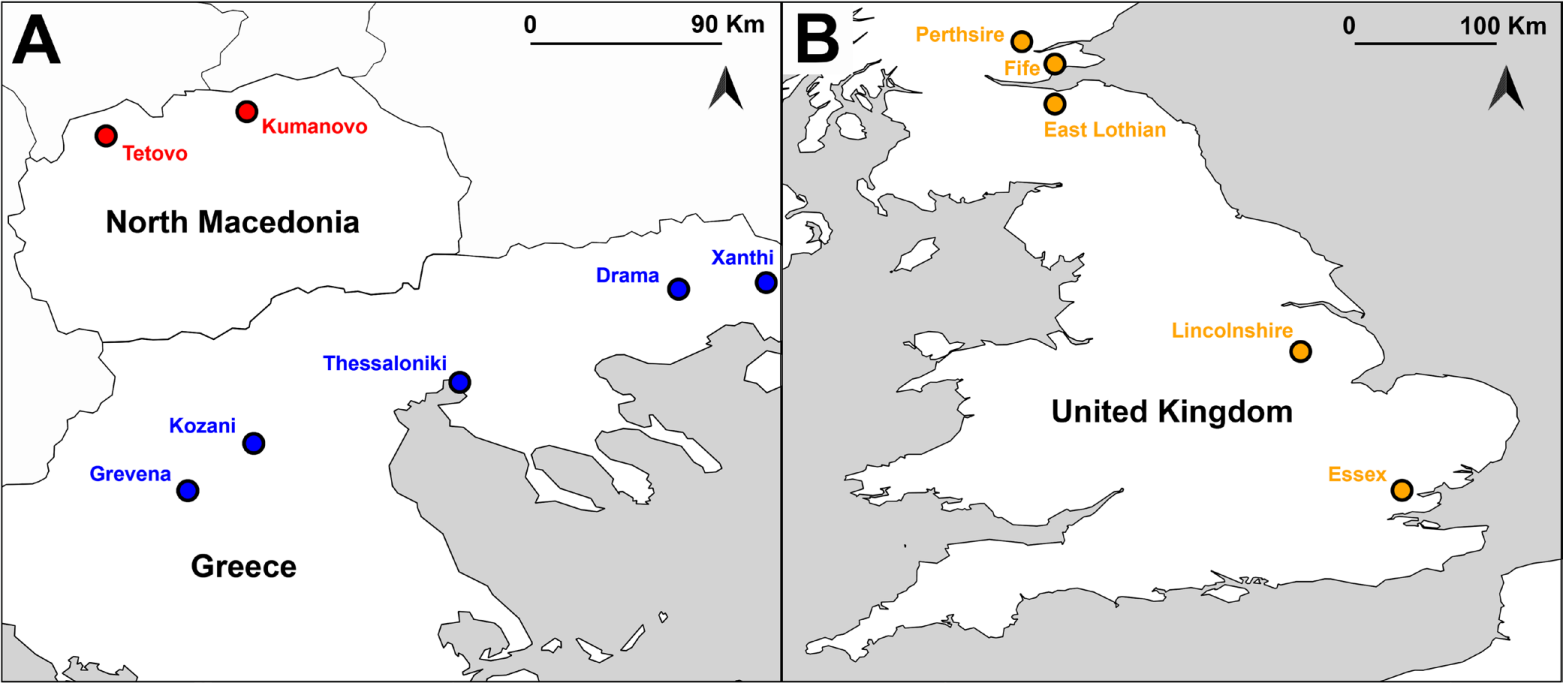
Sampling locations of wild birds in the UK (orange circles) (Part B), Greece (blue circles), and North Macedonia (red circles) (Part A).

A total of 256 samples (blood, muscle tissue, feathers or eggs) were collected from the UK, Greece, and North Macedonia and used in the present analysis (Figure 1). Specifically, 117 samples (61 and 56 of wild and farmed individuals, respectively) from five sampling sites in the UK; 100 samples (74 and 26 of wild and farmed individuals, respectively) from 5 sampling sites in Greece; and 16 samples of wild individuals from two sampling sites in North Macedonia were collected (Table S1). Sampled eggs were obtained from commercial hatcheries; their parents are unknown and were not sampled. A representative sample set from European populations previously published by (Liukkonen‐Anttila et al. 2002) was also provided by the University of Oulu (12 Finnish and 5 Greek individuals) and they were included in our analysis. Furthermore, six mixed‐ancestry individuals from Greece, that is, offsprings of mixed pairing between farmed individuals of Western and wild individuals of Eastern origin, were analyzed. For the remainder of this manuscript, we use the term “hybrid” to define breeding between birds of the Western and Eastern lineage/subspecies of grey partridge.

### 2.2 DNA Extraction

DNA was successfully extracted from all blood/muscle tissue and feather samples either following the DNA extraction protocol by Hillis et al. (1996) based on CTAB reagent for feather and muscle tissue samples or using the QIAamp DNA Mini Kit on blood samples using the manufacturer's protocol. DNA extraction methods were also developed for use with eggs from captive hens. We obtained DNA by locating and dissecting the perivitelline layer from the egg yolk, rinsing it in phosphate‐buffered saline solution and using the QIAamp DNA Investigator Kit to extract DNA. The manufacturer's “Bone and teeth” extraction protocol was followed due to the high calcium content.

### 2.3 DNA Sequencing

Primer pairs LPPGLU (forward)–H414 (reverse) designed to amplify a fragment of the mitochondrial control region (Liukkonen‐Anttila et al. 2002) and L14578 (forward)–H16065 (reverse) of the cytochrome b region (Crowe et al. 2006) were used.

Mitochondrial control region was PCR amplified in a volume of 30 μL, containing 100 ng of genomic DNA, using 1 unit of Qiagen Taq polymerase, 0.2 mM dNTPs, 1 pmol of each primer, 2 mM MgCl_2_, 3 μL of 10× Reaction Buffer, and 0.3 μL of 100× BSA. Thermal cycling amplification conditions were as follows: initial denaturation at 94°C for 2 min, followed by 33 cycles of strand denaturation at 94°C for 1 min, annealing at 54°C for 1 min, and primer extension at 72°C for 1.5 min, with a final 5 min elongation time at 72°C.

For cytochrome b, 100 ng of genomic DNA was amplified in a 25 μL total volume of polymerase chain reaction, using 1 unit of Qiagen Taq polymerase, 0.2 mM dNTPs, 1 pmol of each primer, 1.5 mM MgCl_2_, 3 μL of 10× Reaction Buffer, and 0.3 μL of 100× BSA. Thermal cycling amplification conditions were as follows: initial denaturation at 94°C for 5 min, followed by 35 cycles of strand denaturation at 94°C for 1 min, annealing at 52°C for 1 min, and primer extension at 72°C for 2 min, and a final 8 min elongation time at 72°C. The PCR products were purified using the Nucleospin Extra kit (Macherey‐Nagel, Duren, Germany) and sequenced by the Sanger method using an ABI 3730XL DNA Analyzer.

Although successful in the largest proportion of the samples, DNA extraction from feather samples was often of lower DNA quantity and quality. Targeting these samples, new primers were designed based on the longer sequences obtained from good quality samples to amplify smaller sections of these two loci. Primer3 (https://www.bioinformatics.nl/cgi‐bin/primer3plus/primer3plus.cgi) was used to obtain potential primer pairs amplifying a product of roughly 400 bp. The primers designed for the control region were PER_CR_Frag2F (TGGTTATGCTCGACGTACCA) and PER_CR_Frag2R (ATTCCCCATACACGCAAACC) and those for Cytb were PER_CytB_Frag2F (TGATGAAACTTCGGCTCCCT) and PER_CytB_Frag2R (GGTCGGGTTGTCAACTGAGA).

The shorter control region and cytb fragments were amplified using the same PCR conditions. Reaction volumes were 10 μL, containing 7 μL HotStart PCR master mix (Thermofisher Scientific), 1 μL forward primer (10 μM), 1 μL reverse primer (10 μM) and 1 μL template DNA at 5 ng/μL concentration. Thermal cycling amplification conditions were as follows: initial denaturation at 95°C for 5 min, followed by 40 cycles of strand denaturation at 95°C for 30 s, annealing at 55°C for 30 s, and primer extension at 72°C for 1 min, and a final 10 min elongation time at 72°C.

### 2.4 DNA Sequence Statistical Analysis

A total of 224 sequences of the control region (CR) (Genbank Accession numbers: PQ431575—PQ431798) and 228 sequences of the cytochrome B gene (*cytb*) (Genbank Accession numbers: PQ295559—PQ295786) were aligned using Thompson et al. (1994) and manually trimmed, considering the polymorphic sites detected, as well as the sequencing quality, using the Geneious R10 v10.2.3 software. Thus, the longer sequences obtained using primers by Liukkonen‐Anttila et al. (2002) were reduced substantially in length to align with the smaller amplified products. Data from the present analysis were combined with sequences of *P. perdix* retrieved from GenBank. More specifically, sequences of the main eastern (AF115404) and western (AF115405) haplotypes (Liukkonen‐Anttila et al. 2002), as well as 43 sequences detailed in the Data S1 of that paper were acquired. For the analysis of *cytb*, the sequences AF02879 (Kimball and Braun 2014), EU839466, and GU214276 (Bao et al. 2010) were recovered.

A median‐joining network (Bandelt et al. 1999) was constructed using the software Network 5.0.0 and the frequencies of the sequences (Fluxus Technology) assuming equal weights for all mutations and setting the genetic distance parameter *e* to zero to restrict the choice of feasible links in the final network.

Genetic variability indices of grey partridge populations, that is, the number of distinct haplotypes (*h*), haplotype diversity (Hd) and nucleotide diversity (*π*) values, were estimated using the combined data set of both CR and cytb with dnasp 5 (Librado and Rozas 2009) software.

### 2.5 Microsatellite Amplification

Eight microsatellite loci were analyzed by multiplexing in two independent reactions (four loci per reaction—Table S2). Multiplex PCRs were performed in 10 μL reactions using 5 μL of Qiagen Buffer from the Qiagen multiplex PCR kit and 30 ng of genomic DNA. Primer concentration for each amplified locus is given in Table S2. PCR amplification was performed using a TD 60–50 protocol that was set according to Table S3. Microsatellite analysis was performed in an ABI 3130L Genetic Analyzer (Applied Biosystems) and genotypic data were obtained using the Geneious R.v11 software.

### 2.6 Microsatellite Statistical Analysis

Observed (*H*_o_) and expected (*H*_e_) heterozygosity values for each locus were calculated using GENEPOP 4.0 (Rousset 2008). Deviation from Hardy–Weinberg (HW) equilibrium was tested using Fisher's exact tests (Rousset 2008) with the same software. FSTAT 2.9.3.2 (Goudet 1995) was used to compute inbreeding coefficient values (*F*_IS_) and allelic richness (*A*_R_). CERVUS 3.0.3 (Kalinowski et al. 2007) was used to evaluate polymorphic information content (PIC), null allele probability, and the mean number of alleles for each locus. *F*st values between pairs of groups were calculated using Fstat 2.9 software (Goudet 1995).

The genetic structure of grey partridge was investigated using a Bayesian clustering method. STRUCTURE 2.3 (Pritchard et al. 2000) was used to infer the number of genetic clusters (*K*). The log‐likelihoods of our data set (ln Pr [X|K]) were estimated for different numbers of genetic clusters using an admixture ancestry model based on 100,000 burn‐in steps followed by 1,000,000 MCMC replicates. We utilized a method developed by (Evanno et al. 2005) to determine the number of populations present, based on the second‐order rate of change in the log probability of the data (*ΔK*) among 20 runs of each assumed *K* using the web‐based utility “Harvest” (http://taylor0.biology.ucla.edu/struct_harvest).

### 2.7 Molecular Sex Identification

For the molecular sex identification in the grey partridge, conserved primers were used, namely the 1237 L—1272H pair (Kahn et al. 1998) which delineates an intron that varies in size between the two sex chromosomes in most bird species. The total volume of the polymerase chain reaction was 10 μL in which 100 ng of genomic DNA was amplified, using 0.5 unit of Qiagen Taq polymerase, 0.2 mM dNTPs, 1 pmol of each primer, 1.5 mM MgCl_2_, and 1 μL of 10× Reaction Buffer. Thermal cycling amplification conditions were as follows: initial denaturation at 95°C for 5 min, followed by 34 cycles of strand denaturation at 94°C for 45 s, annealing at 56°C for 45 s, and primer extension at 72°C for 1 min, and a final 7 min elongation time at 72°C.

### 2.8 ddRAD Library Preparation

DNA quality was assessed via agarose gel electrophoresis on a 1% gel, and only nondegraded DNA (as judged by a tight high molecular weight band against a lambda standard) was selected for the library preparation stage. DNA was quantified using a Qubit Broad Range dsDNA Assay (ThermoFisher Scientific) according to the manufacturer's instructions and normalized to c. 7 ng/μL.

A total of 2 ddRAD libraries were constructed according to a modified protocol of the original (Peterson et al. 2012) methodology. This is described in detail elsewhere (Brown et al. 2016; Manousaki et al. 2016). An inter‐and intra‐library replicate sample was included during the construction of each library. Briefly, for each library, individual genomic DNA (21 ng per sample) was restriction digested by *Sbf*I and *Sph*I, and then Illumina‐specific sequencing adaptors (P1 & P2), each with unique combinatorial inline barcodes, were ligated to fragment ends. The samples were then pooled and size selected (400–700 bp fragments) by gel electrophoresis, PCR amplified (15 cycles) and the resultant amplicons (ddRAD library) were purified and quantified. The combinatorial inline barcodes (5 or 7 bases long) included in the P1 and P2 adaptors allowed each sample replicate to be demultiplexed post‐sequencing. Each ddRAD library was sequenced on the Illumina MiSeq Platform (a single paired‐end run; v2 chemistry, 2 × 150 bases).

### 2.9 ddRAD SNP Calling

The sequences were quality assessed using FastQC (Andrews 2010) and the reads were demultiplexed by barcode using the “*process_radtags*” module (default parameters) of the Stacks v1.48 bioinformatics pipeline (Catchen et al. 2013). This module also filtered out low‐quality reads. The retained reads, now missing variable‐length barcodes, were then trimmed to a standard 148 bases in length. For each individual, matching forward and reverse reads were then concatenated into a single longer “artificial” read using a custom Perl script (Genbank BioProject Accession number: PRJNA1173582). Individuals that had < 100,000 reads were removed from further processing.

The individual data were then processed using the “*denovo_map.pl*” module of stacks (‐m 10 ‐M 2 ‐n 0) to assemble and create a catalog of genetic loci contained in the data. The Stacks scripts “*export_sql.pl*” and “*populations*” and filtering steps were used to retain all loci that fulfilled the following criteria: (1) contained exactly one SNP (in the concatenated forward and reverse reads) to avoid physically linked markers and ensure conserved sequence surrounding the target SNP to facilitate primer design; (2) contained exactly two alleles; (3) had a read depth of ≥ 10 reads per individual to maximize the likelihood of the SNP being real.

### 2.10 ddRAD Statistical Analysis

A genotype file was created containing the SNP genotypes, called from the ddRAD data, for 87 individuals and 8236 SNPs. All the SNPs were checked and were biallelic, and we proceeded with the filtering of the dataset. The PLINK toolbox (Purcell et al. 2007) was used to remove loci that were not genotyped in ≥ 80% of the individuals. Additionally, a dataset containing three full‐sib families (parents and two offsprings) was used to check the mendelian inheritance of the alleles.

The genetic diversity indices as well as a Principal Component Analysis (PCA), based on Nei's genetic distance (Nei et al. 1983) were calculated using GENEALEX 6.502 software (Peakall and Smouse 2006). Population pairwise FST was calculated using Arlequin 3.5 (Excoffier and Lischer 2010). The genetic structure of the grey partridge was investigated using STRUCTURE 2.3 (Pritchard et al. 2000). The log likelihoods of our data set (ln Pr [X|K]) were estimated for different numbers of genetic clusters using an admixture ancestry model based on 100,000 burn‐in steps followed by 1,000,000 Markov chain Monte Carlo (MCMC) replicates. We utilized a method developed by (Evanno et al. 2005) to determine the number of populations present, based on the second‐order rate of change in the log probability of the data (*Δ*K) among 20 runs of each assumed K using the web‐based utility “Harvest” (http://taylor0.biology.ucla.edu/struct_harvest). We then evaluated the membership coefficient value (*q*) for each individual.

Analysis for the identification of an informative SNP marker panel for allowing population assignment and identification of non‐native stocks for captive rearing was performed based on the “Informativeness of assignment” method, as implemented in TRES software (Kavakiotis et al. 2015). Furthermore, assignment tests were performed according to Rannala and Mountain method (Rannala and Mountain 1997) in GENECLASS2 software (Piry et al. 2004).

## 3 Results

### 3.1 MtDNA Analysis

Aligned sequences of a 381 bp section of the control region (CR) (*n* = 224 individuals) and a 370 bp section of the cytochrome B gene (*cytb*) (*n* = 228 individuals) were obtained. The CR was more polymorphic, revealing 21 haplotypes compared to cytb, where only five haplotypes were detected. A total of 204 individuals have been sequenced at both regions, and the mitochondrial diversity estimates of the combined sequences (751 bp in total) are summarized in Table 1. A total of 24 haplotypes (*h*) were observed, with 11 found within the wild UK birds and six within the wild Greek birds. Our dataset revealed that there was greater mitochondrial diversity in the wild UK birds (haplotype diversity = 0.776) than in the wild Greek birds (haplotype diversity = 0.439). This pattern was also mirrored in the captive stocks of both countries, with the UK captive stocks (hd = 0.786) having a higher diversity than the Greek captive stocks (hd = 0.409). Surprisingly, the wild‐caught birds from North Macedonia had the highest haplotype diversity (hd = 0.833). The three sampling locations with the highest haplotype diversity were all from different countries: East Lothian (hd = 0.868; Scotland), Kumanovo (hd = 0.822; North Macedonia) and Kozani (hd = 0.757; Greece). This is contrasted with the two lowest estimates, also from different countries: Fife (hd = 0.182; Scotland) and Drama (hd = 0; Greece).

**TABLE 1:** Summary of mitochondrial diversity estimates based on both gene regions (751 bp). Estimates have only been calculated for locations where ≥ 10 individuals were sampled.

Region	Locations	No. individuals	*H*	Hd	Hd (SD)	*π*	*S*
UK_wild	ALL (*n* = 5)	42	11	0.776	0.053	0.00168	10
	Fife	11	2	0.182	0.144	0.00024	1
	East lothian	26	10	0.868	0.036	0.00238	9
UK_captive	ALL (*n* = 5)	44	6	0.786	0.026	0.00161	5
Greece_wild	ALL (*n* = 5)	73	6	0.439	0.069	0.00107	8
	Drama	30	1	0	0	0	0
	Grevena	13	3	0.603	0.088	0.00113	3
	Kozani	17	5	0.757	0.063	0.00239	7
Greece_captive	ALL (*n* = 2)	29	3	0.409	0.102	0.00076	2
North Macedonia_wild	ALL (*n* = 2)	13	6	0.833	0.071	0.0095	21
	Kumanovo	10	5	0.822	0.097	0.00936	21
ALL	20+	201^a^	24	0.838	0.014	0.0118	37

Abbreviations: H, No. of haplotypes; Hd (SD), Hd Standard deviation; Hd, Haplotype diversity; S, No. of variable sites; π, nucleotide diversity.

^a^ Three Finnish samples analyzed in both genes were not included in the analysis.

**FIGURE 2:**
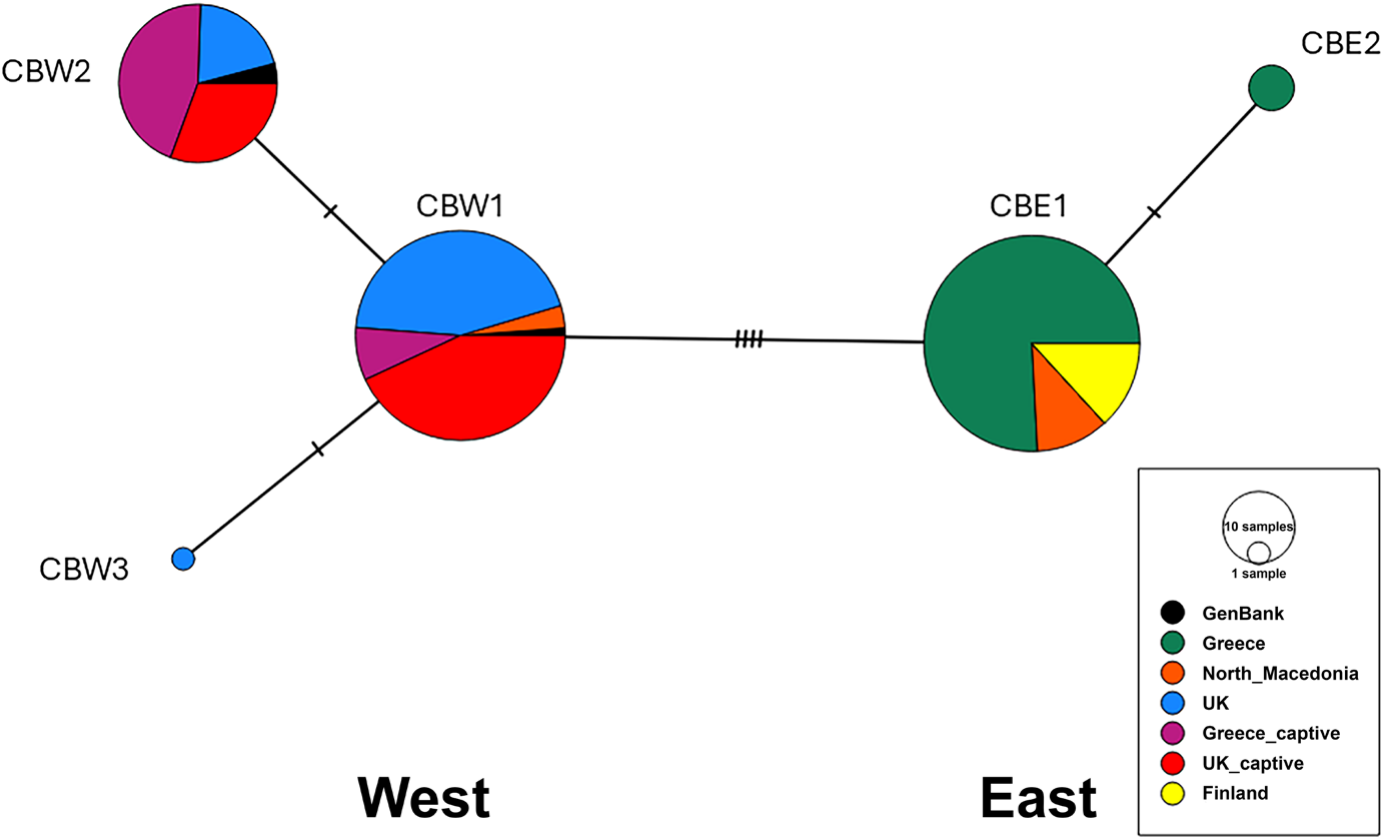
Haplotype network of the Cyt‐B sequences, including three *Perdix perdix* Genbank sequences. Each circle represents a distinct haplotype or maternal lineage, and the markers on the lines between them represent the number of base‐pair differences between each lineage.

**FIGURE 3:**
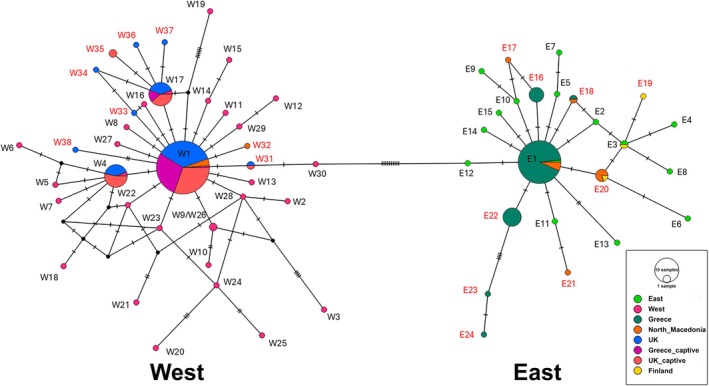
Haplotype network of the CR sequences including the Eastern (E) and Western (W) haplotypes of Liukkonen‐Anttila et al. (2002). Each circle represents a distinct haplotype or maternal lineage and the markers on the lines between them represent the number of base pairs between each lineage. The new haplotypes identified in this study are numbered in red.

The haplotype networks revealed and confirmed the eastern and western lineages in both the cytb and CR sequences (Figures 2 and 3). The inclusion of the control region haplotypes by (Liukkonen‐Anttila et al. 2002) in our network verified the structuring of our samples into the Western and Eastern lineages, since all wild birds in the UK had a western mitochondrial haplotype, while all wild birds in Greece showed an Eastern haplotype. The two populations from North Macedonia had a mix of birds as indicated by the network based on the CR data; most of the birds had an eastern haplotype (69%, *n* = 11) and some possessed a western haplotype (31%, *n* = 5). It should be noted that the control region haplotype network has a larger number of nodes and therefore a more complex evolutionary history that could not be fully resolved. This is a consequence of the higher mutation rate of this gene region compared to cytb and could potentially be resolved by including additional samples from other geographic localities to better reflect past evolutionary processes.

The analysis also revealed that 17 new haplotypes (8 western and 9 Eastern) exist within our control‐region dataset that were not found in the previous study. The most striking observation is the clustering of all Greek captive individuals within the Western lineage, showing higher relatedness with the wild birds from the UK, rather than those in Greece.

### 3.2 Microsatellite Analysis

In total, 106 grey partridge samples from the UK and 118 samples from Greece/North Macedonia were used for microsatellite analysis at eight loci. All samples that were successfully genotyped in more than six loci were included in the global analysis. Thus, a dataset was created with 224 individuals, and they were split into five different groups for further analysis: “UK Wild” including all individuals from Scotland and England, “UK Captive”, “Greek/North Macedonia Wild”, “Greek Captive” referring to Greek farmed individuals and “Hybrids” first generation offspring of Greek captive individuals paired with wild individuals. Eighty alleles were found across the eight microsatellite loci. The most variable loci were MNT412 and MNT12, while the least polymorphic were MNT404, MNT45 and Aru1A (Table S4).

Genetic diversity indices were estimated for the five different groups of samples (Table 2). Heterozygosity values were moderate for all groups, ranging from 0.438 to 0.631, with captive stocks, as expected, showing higher Fis values due to inbreeding. None of the populations met the criteria for HWE at a significance level of *p* ≤ 0.05. Further analysis revealed that most loci were out of HWE, probably due to null alleles (frequencies 15%–20%). Pairwise *F*_ST_ values range from 0.069 (UK Captive‐Greek Captive) to 0.2 (Hybrids‐UK Wild).

**TABLE 2:** Genetic diversity indices per group of samples analyzed for eight microsatellite markers (left column) and 2300 SNPs (right column).

Sample origin	*Ν*	*A*_r_	*H*_e_	*H*_O_	*F*_is_	*P*_HW_
UK wild	57/16	3.38/1.20	0.59/0.13	0.55/0.12	0.07/0.035	0.06/0.9
UK captive	49/3	3.64/1.15	0.63/0.09	0.55/0.12	0.13/−0.32	0/0
Greece/North macedonia wild	95/43	3.69/1.22	0.57/0.14	0.46/0.12	0.08/0.05	0/0.9
Greek captive	17/14	2.90/1.21	0.55/0.13	0.51/0.12	0.19/−0.02	0/0.9
Hybrids	6/5	3.07/1.19	0.47/0.12	0.42/0.16	0.04/−0.17	0.75/0

Abbreviations: *A*_r_, mean allelic richness; *F*_is_, inbreeding coefficient; *H*_e_, expected heterozygosity; *H*_o_, observed heterozygosity; *P*_HW_, *p*‐value of Hardy–Weinberg; *Ν*, number of analyzed individuals.

**FIGURE 4:**
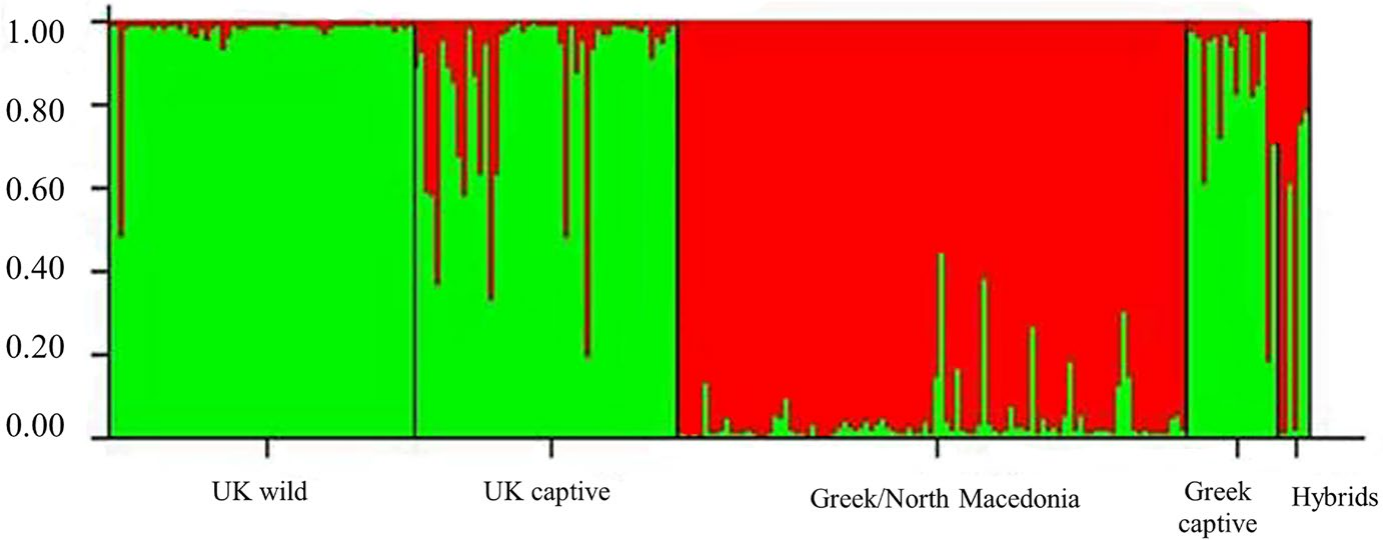
A genetic structure plot of the global microsatellite database, consisting of 8 microsatellites. Each bar represents an individual bird and the proportion of green color represents the proportion of “western” genetic ancestry, and the proportion of red represents the proportion of “eastern” genetic ancestry.

Population genetic structure was investigated on the entire global dataset and then separately for the wild populations. In the global analysis, there was a clear separation into two clusters, geographically into Western and Eastern lineages (Figure 4) where wild birds of North Macedonia and Greece clearly cluster separately from the wild birds of the UK. However, Greek captive stocks are more closely aligned with the UK wild birds than any of the wild Greek birds. The microsatellites also reveal finer‐scale information (compared to mitochondrial data), including the large number of individuals within the captive UK stocks that appear to descend from birds of mixed (Western and Eastern) origin, though predominantly Western. Small evidence of hybrid individuals was even found in the wild birds (one in the UK and approximately five in Greece/North Macedonia).

No genetic structure was found within the wild UK birds (Figure S1). Regarding the population structure of wild birds in Greece and North Macedonia, three main geographical clusters were revealed by the analysis (Figure S2). One genetic cluster consisted of the birds from Drama in north‐eastern Greece, the second genetic cluster included birds within North Macedonia, and the third cluster included mostly birds from the Grevena region. Birds in Grevena and the three remaining populations (Kozani, Thessaloniki and Xanthi) exhibited a mix of the three genetic clusters, with the most south‐westerly population (Grevena) showing slight distinction, that is, exhibiting less genetic similarity to the birds from Drama than the other populations.

### 3.3 ddRAD Library

A ddRAD library was generated for 87 individuals. This dataset consisted of 8329 SNPs. These were subsequently filtered into those SNPs that had been genotyped in > 80% of the 87 individuals, leaving a final dataset of 2300 SNPs. Additionally, one individual was run in triplicate to check data consistency. Only a few discrepancies in the genotypes (0.26%) were detected, including mainly some missing genotypes and some allelic dropouts. Only a few instances of non‐Mendelian inheritance (0.9%) were detected using the datasets from the known related families. The juveniles from families were subsequently discarded from further analysis, leaving us with a dataset of 81 individuals.

The sampled individuals were grouped in the same five population clusters as in the microsatellite analysis, and the genetic diversity indices are presented in Table 2. Estimates of heterozygosity values were much lower compared to those obtained from microsatellite data, while inbreeding coefficient levels were close to zero or negative for all populations. Pairwise *F*_ST_ values range from 0.054 (UK Captive—Greek Captive) to 0.24 (Greece/North Macedonia—UK Captive).

**FIGURE 5:**
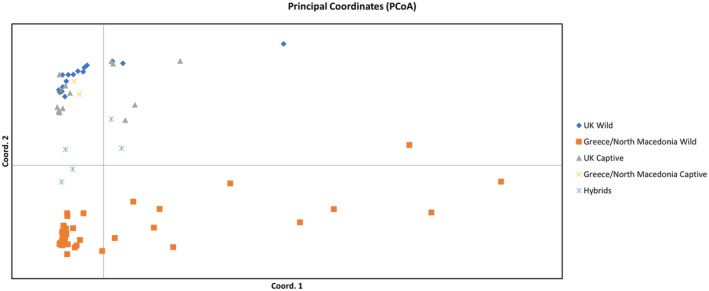
Principal Component Analysis (PCA) of the 81 individuals based on 2300 SNPs.

**FIGURE 6:**
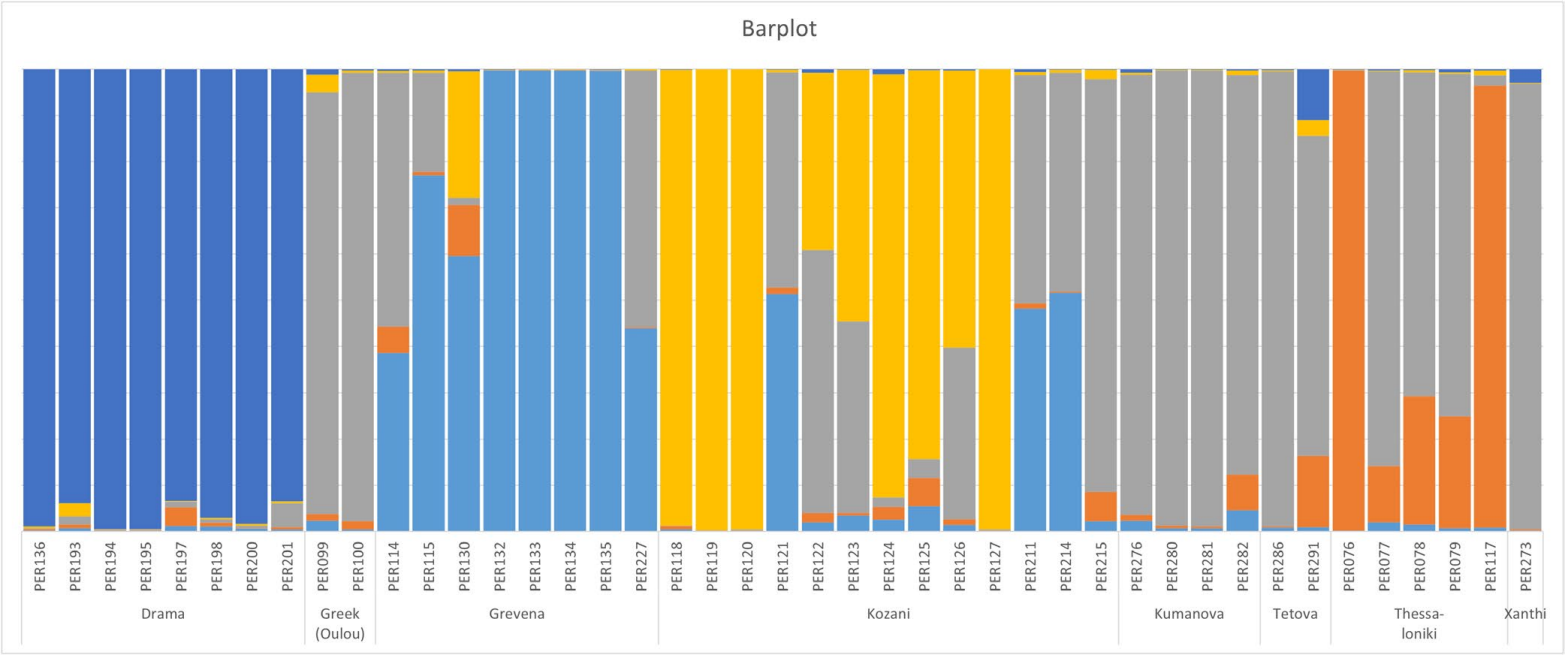
Genetic structure plot of the wild‐caught Greek and North Macedonia birds based on 2300 SNPs. Each bar represents an individual bird, and the proportion of each color represents the proportion of each of five genetic ancestries. The locations refer to those shown in Figure 1.

Principal Component Analysis (PCA) on the SNP data revealed two main phylogeographic superclusters (Figure 5). The UK Wild clustered with all Captive samples, while the Greek/North Macedonia Wild clustered separately. Further analyses within the two main superclusters did not provide strong resolution for the UK cluster, since only 16 individuals were successfully genotyped using the 2300 SNPs. There was evidence of two genetic lineages that occur across all individuals, but with a slightly higher representation of one of them in the birds from East Lothian (data not shown). As regards the East‐Wild populations, ddRAD data provide strong evidence for five genetic lineages (Figure 6) (Drama, Grevena, Kozani, North Macedonia, and Thessaloniki). The most distinct were the birds from Drama. The Thessaloniki group showed the lowest levels of distinctiveness, with some individuals aligning closely with the North Macedonia group. Also, similar to the microsatellite data, the individuals from Kozani show marked diversity, with some individuals showing similarities to the Grevena and North Macedonia populations. This aligns with the geographic position of Kozani, between Grevena and the Macedonian border (Figure 6).

**FIGURE 7:**
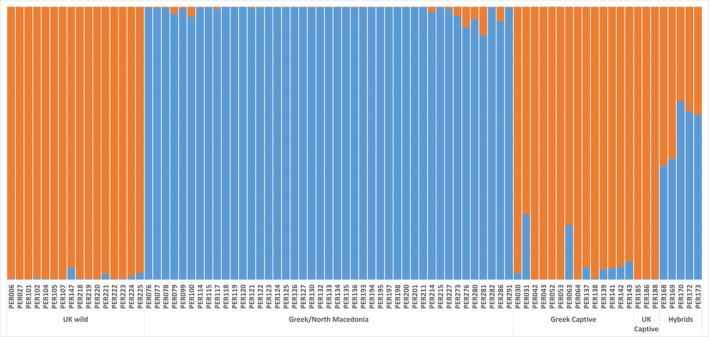
Genetic structure plot focusing on the levels of hybridization in the sampled Grey partridges using 2300 SNPs. Each bar represents an individual bird and the proportion of orange color represents the proportion of “western” genetic ancestry, and the proportion of blue represents the proportion of “eastern” genetic ancestry.

Further analysis was focused on the ability of the SNP dataset to differentiate hybrids and evaluate individuals from each population cluster. Based on the presence of two superclusters (West/East), we examined the assignment of the individuals in these two groups (Figure 7). Hybrid samples are indeed clearly detected, while a minor hybridization level is present in other individuals, such as the Greek Captive.

Analysis for the identification of an informative SNP marker panel, for allowing population assignment and identification of non‐native stocks for captive rearing, was further performed. All 2300 SNPs were scored for their informativeness to distinguish an individual from either the UK Wild or the Greek/North Macedonia Wild population cluster. The 15 top‐scoring SNPs were selected; alleles and genotype frequencies are shown in Table S5. In order to test the identification strength, assignment tests were performed by splitting the dataset randomly into reference and test datasets with a 70/30 ratio. All the tested individuals were assigned successfully to their population of origin. The 15‐SNP panel identified the origin (West/East) of the individuals accurately with a score of 100%.

## 4 Discussion

### 4.1 Phylogeography of Grey Partridge

The phylogeographic data produced by (Liukkonen‐Anttila et al. 2002) were used as a basis for comparisons with our dataset. We sequenced an overlapping section of the mitochondrial control region that (Liukkonen‐Anttila et al. 2002) sequenced in individuals sampled across 17 Eurasian countries. One of their main findings was that there was an Eastern/Western split in mitochondrial haplotypes within Europe, suggesting that grey partridge had colonized Europe from two separate glacial refugia. However, only a few samples had been collected in Greece and none within Scotland. In our analysis, we have included a large number of data from these two countries as well as North Macedonia, and we found that all wild birds in the UK had a western mitochondrial haplotype. Also consistent with (Liukkonen‐Anttila et al. 2002), all studied wild birds in Greece belong to the Eastern lineage. The fact that the two populations from North Macedonia had a mix of birds matches the situation in neighboring Bulgaria, perhaps suggesting that these regions of the Balkans are contact zones between the eastern and western lineages (Liukkonen‐Anttila et al. 2002). Alternatively, birds of Western origin may have been released for hunting. However, more research focusing on the Balkans is required to distinguish between these two scenarios.

By comparing the mitochondrial haplotypes to those previously published, we can also gain some limited insight into the origins of the lineages. Most of the wild birds in the UK had the main western haplotype (W1). This occurs throughout Europe in wild and captive stocks (Liukkonen‐Anttila et al. 2002; Andersen and Kahlert 2012). However, the two other main haplotypes in our UK samples (W17 and W4) had also been previously identified by (Liukkonen‐Anttila et al. 2002) (Figure 3). The haplotype W17 was found in this study in captive birds from Greece and both captive and wild from the UK. A Danish study concluded that this haplotype originates from Bohemia (Czechia) and is found in European captive stocks and Danish populations that were stocked with Bohemian birds between 1895 and 1934 (Andersen and Kahlert 2012). The captive origin of this haplotype is consistent with our data. The third haplotype that is most common in the UK samples is W4; this haplotype is highly likely to be of French origin based on the study by (Liukkonen‐Anttila et al. 2002). The other haplotypes revealed in our British samples are novel; however, the majority are very similar to the bohemian haplotype (W17). Neither of the two haplotypes that were unique to British wild birds in the previous study was found among our samples. As far as the origin of lineages in Greece, the main eastern haplotype (E1) that has been previously described in Finland, Bulgaria, and Ireland (Liukkonen‐Anttila et al. 2002), was the most frequent and widespread haplotype in Greece, and it probably originated and expanded from a Balkan refugium. Five out of seven haplotypes detected in Greece were novel, with haplotypes E22 and E20 being the second and third most abundant, respectively. North Macedonia consisted of the two most frequent haplotypes in Europe (E1 and W1), haplotype E16 with a probable Finnish origin, and four new haplotypes.

The mitochondrial data is, therefore, of limited use in population assignment; we can accurately use it to identify the presence of Eastern and Western lineages, but without a much larger database of historical samples, it is currently difficult to identify the geographic origins of each haplotype, as the majority of British wild birds have haplotypes that are found in captive stocks.

### 4.2 Power of Different Types of Markers to Detect the Population Structure

When comparing the relative performance of microsatellites and RADseq, there was the expected discrepancy of higher heterozygosity values in the microsatellite compared to the RADseq data, which reflect the high mutational rate of microsatellites. Overall, both markers were able to uncover genetic structuring. Based on the small but widely geographically spread number of sampling sites in the UK, both microsatellite and SNP data agree that there is a lack of deep phylogeographic structure within the wild British samples. Considering the larger distance between sampling sites compared to Greek sampling sites, for example, more than 500 km between Fife and Essex, this was unexpected. This result is suggestive of a large, homogenous population within the UK, but considering the low dispersal of the grey partridge, this points toward large‐scale human‐mediated translocations. However, sampling from a much larger number of sites with known histories of management and partridge release is required to confirm this finding more generally. On the contrary, both molecular markers suggested similar trends concerning phylogeographic structure in Greek populations, with SNP data being somewhat more conclusive, although fewer individuals were included in the analysis. The most distinct was the population of Drama, followed by the population of Grevena, while the other three populations (Kozani, Thessaloniki and Xanthi) revealed more mixed patterns and greater similarity with North Macedonia.

To be able to confidently assign individuals to either a Western or Eastern origin, a marker panel of 15 SNPs has been identified, based on the ddRAD libraries. Only wild individuals were used for this panel development; however, not a single SNP was at fixation in both populations, that is, only occurred in the Eastern individuals while completely absent from the Western individuals (see Table S5 _for a summary of the SNP frequencies). These may reflect either the recent divergence of the two lineages from the common ancestor or could be the result of past introduction events that affected allele frequencies. However, this panel of 15 SNPs could be combined with a mitochondrial DNA test for the identification of non‐native stocks within Greece or within the UK.

### 4.3 Effect of Introgressive Hybridization on Wild Populations

All three datasets (mitochondrial, microsatellites and SNPs) show that captive stocks in both the UK and Greece have predominantly derived from birds of western origin ( *Perdix perdix perdix* ). However, there is also evidence of Eastern genotypes within both of these captive populations, signaling that birds of the eastern lineage ( *Perdix perdix lucida* ) have also been used for captive breeding. However, considering that in both the UK and Greece, individuals from captive stocks are released each year, very little evidence of hybrids within the wild individuals has been found; one hybrid individual was identified in the UK and five in Greece/North Macedonia based on microsatellites (Figure 4). Although they provide insight, the use of just eight microsatellites has limitations. As can be seen with the known hybrid individuals, only three out of the six can be identified as hybrids using this microsatellite data. However, even when considering the robust SNP data, there is no evidence of F1 hybrids in the wild (Figure 7), although the microsatellite data indicates that there may be one wild F1 hybrid in the UK dataset (Figure 4).

Obviously, Greece would be predicted to have a larger number of hybrids, considering the captive stocks being released are of Western origin; however, there is very little evidence to suggest that hybridization has occurred within the wild Greek birds. This contrasts with neighboring North Macedonia, where all our genetic datasets (mitochondrial, microsatellite and SNPs) indicate that individuals of both eastern and western genealogies are present in the wild population. However, as already discussed, whether this is a natural transition zone between *P. p. perdix* and *P. p. lucida* or the result of captive releases requires further investigation. The differences between levels of hybridization in the captive stocks compared to the wild individuals in both the UK and Greece are intriguing. Several reasons could explain this: (1) Released birds do not survive and therefore do not contribute to the wild gene pool, (2) Released hybrid individuals do not survive and do not contribute to the wild gene pool, (3) Hybridization within the captive stocks is a relatively recent phenomenon, and not many hybrids have been released yet, (4) The numbers of released birds have been very small compared to the wild population. We argue that the last point is unlikely, as the grey partridge has been the most important European game bird for centuries, and there is historical evidence that stocking and translocations have been conducted throughout Europe as far back as the 1560s (Andersen and Kahlert 2012). The other common name of the grey partridge (the Hungarian Partridge) is based on the widespread exporting of captive stock from Hungary across Europe and to North America during the 20th century (Browne et al. 2009). Estonian and French birds were more recently imported into Ireland for captive breeding between 2002–2005 (Buckley et al. 2012). This widespread sharing of game bird stock across Europe is of routine and historical occurrence. However, the geographical origins of captive stock are rarely known. Regarding the third point, it does seem that captive populations are mainly of Western origin. This has been the finding of our study in both the UK and Greece but also of the two previous genetic studies in Finland and Denmark (Liukkonen‐Anttila et al. 2002; Andersen and Kahlert 2012). It is, therefore, feasible that hybridization is of limited success or has not occurred until recently. However, regarding the first two points, multiple studies have found that released birds have very low survival (Brittas et al. 1992; Mallord et al. 2024). This is the most likely explanation for Greece, where releases of captive birds have occurred since the 1980s, but without evidence of western genotypes in the wild populations yet. However, contrasting with Greece, in the UK, we failed to find evidence of genetic structure between wild populations within the UK. These wild populations also share genetic similarities to the captive population based on mitochondrial (Figure 3) and microsatellite data (Figure S3). Considering the low dispersal of Grey Partridge, this is indicative of widespread historic translocation of birds and/or the survival of captive releases.

Future work will benefit from the use of museum samples to gain insights on the origin of all studied wild grey partridge populations. A similar study conducted on Italian museum samples (Greco et al. 2024) revealed that large‐scale reintroductions had adverse genetic effects on wild populations, causing the indigenous gene pool to almost disappear. Unlike in Greece, we found very little population structure in UK populations, suggesting that UK birds are the result of large mixing of different populations due to stocking with allocthonous individuals. To determine whether there are remnants of historical populations, the use of museum reference samples will be of great importance.

## 5 Conclusions

This study has confirmed, in accordance with previous studies, two main grey partridge lineages within Europe, termed Eastern and Western. In the UK and Greece, the captive grey partridge used for release is mostly of Western heritage; however, the respective wild birds of each country are of differing ancestries, west and east, respectively. Our results suggest there has been differing success of released birds in these two locations. In the UK, the wild birds share mitochondrial similarities with the captive stocks; however, they show very little introgression of the eastern lineage, considering it is present in UK captive stocks. In Greece, the success of released stock is even weaker, with very little evidence that any of the captive stock has bred successfully after release. Since releases using captive stock have been ongoing for decades, these results highlight the need for considering the heritage of captive birds used in releases if the aim is to bolster populations for long‐term conservation success. We advise that further research aims to test whether this finding is due to the overall low success rate of released birds generally or outbreeding depression due to unique adaptations in the two lineages. We also suggest further work on museum samples would be beneficial, as the long history of grey partridge releases in Europe may have already drastically changed the genetics of the wild‐living birds. This could be an explanation for the low population structure in the UK compared to Greece, potentially being caused by a history of stocking with allochthonous individuals.

## Author Contributions

**N. Karaiskou:** data curation (equal), formal analysis (equal), investigation (equal), methodology (equal), supervision (equal), visualization (equal), writing – original draft (equal), writing – review and editing (equal). **N. Manios:** resources (equal). **D. Kiousis:** resources (equal). **E. Chatzinikos:** resources (equal), writing – review and editing (supporting). **J. C. Dunn:** resources (equal). **A. Triantafyllidis:** supervision (lead), writing – review and editing (supporting). **D. Parish:** resources (equal), writing – review and editing (supporting). **A. Moulistanos:** data curation (supporting), investigation (supporting), writing – review and editing (supporting). **K. Gkagkavouzis:** data curation (supporting), formal analysis (equal), methodology (supporting), project administration (supporting), visualization (equal), writing – original draft (supporting), writing – review and editing (supporting). **A. Ball:** data curation (equal), formal analysis (equal), investigation (equal), methodology (equal), supervision (equal), visualization (equal), writing – original draft (supporting), writing – review and editing (equal). **K. Kalaentzis:** data curation (supporting), investigation (supporting), visualization (supporting), writing – review and editing (supporting). **S. Minoudi:** investigation (supporting). **G. Murray‐Dickson:** data curation (equal), formal analysis (equal), methodology (equal), writing – review and editing (equal). **M. Ghazali:** formal analysis (supporting), investigation (supporting).

## Conflicts of Interest

The authors declare no conflicts of interest.

## Supporting information


**Figure S1.** A genetic structure plot of the wild‐caught UK individuals using data from 8 microsatellites. Each bar represents an individual bird and the proportion of each color represents the proportion of different ancestry. The plot shows that all the birds have very similar ancestry. The sampling locations are those shown on the map in Figure 1.

**Figure S2.** A genetic structure plot of the wild‐caught Greek and North Macedonia individuals using the data from eight microsatellites. Each bar represents an individual bird and the proportion of each color represents the proportion of the different genetic ancestry. Three population clusters were present. The sampling locations are those shown on the map in Figure 1.

**Figure S3.** Genetic Structure plot of all birds sampled within the UK. Each bar represents an individual bird and the proportion of each color represents the proportion of the two genetic ancestries. There is very little differentiation with only two clusters. The one incorporates the Scottish and Northern Irish captive (C) individuals (blue) while the other, most of the wild‐caught individuals, along with the captive Suffolk population (green).

Data S1
**Table S1.** Sampling information of all analyzed samples: sample id, CR haplotype for each sample and Accession number, Cyt‐b haplotype for each sample and Accession number, number of microsatellite loci analyzed per sample, samples were ddRAD sequences were obtained, wild or captive, country of origin, sampling region, type of biological material and sex of all analyzed samples.
**Table S2.** Primer information and primer concentration of the eight microsatellite loci used in the two multiplex PCR reactions.
**Table S3.** PCR amplification conditions of multiplex PCR reaction for microsatellite analysis
**Table S4.** Microsatellite loci analyzed. Number of alleles (A), allelic size range in base pairs (R), expected and observed heterozygosity (He, Ho), probability value for Hardy–Weinberg tests (P_HW_), polymorphic information content (PIC), null alleles per locus (F), sample size (N), mean number of alleles (Amean), and allelic richness (A_R_) for all samples.
**Table S5.** Allele and genotype frequencies for the top‐scored 15 SNPs.

## Data Availability

A total of 224 sequences of the control region (CR) (Genbank Accession numbers: PQ431575—PQ431798) and 228 sequences of the cytochrome B gene (cytb) (Genbank Accession numbers: PQ295559—PQ295786) were produced in this work and were deposited in a public database. Regarding the ddRAD data, for each sample, matching forward and reverse reads were concatenated into a single longer “artificial” read, and they were all deposited in a public database (Genbank BioProject Accession number: PRJNA1173582).
