# Supplementary figures and images for: Grey Partridge (Perdix perdix) Introductions: Genetic Survey on Wild and Captive Populations at the Edges of the Range
Source: Ecol Evol. 2025 Mar 21;15(3):e71122. doi: 10.1002/ece3.71122 (PMC11926436; doi:10.1002/ece3.71122)

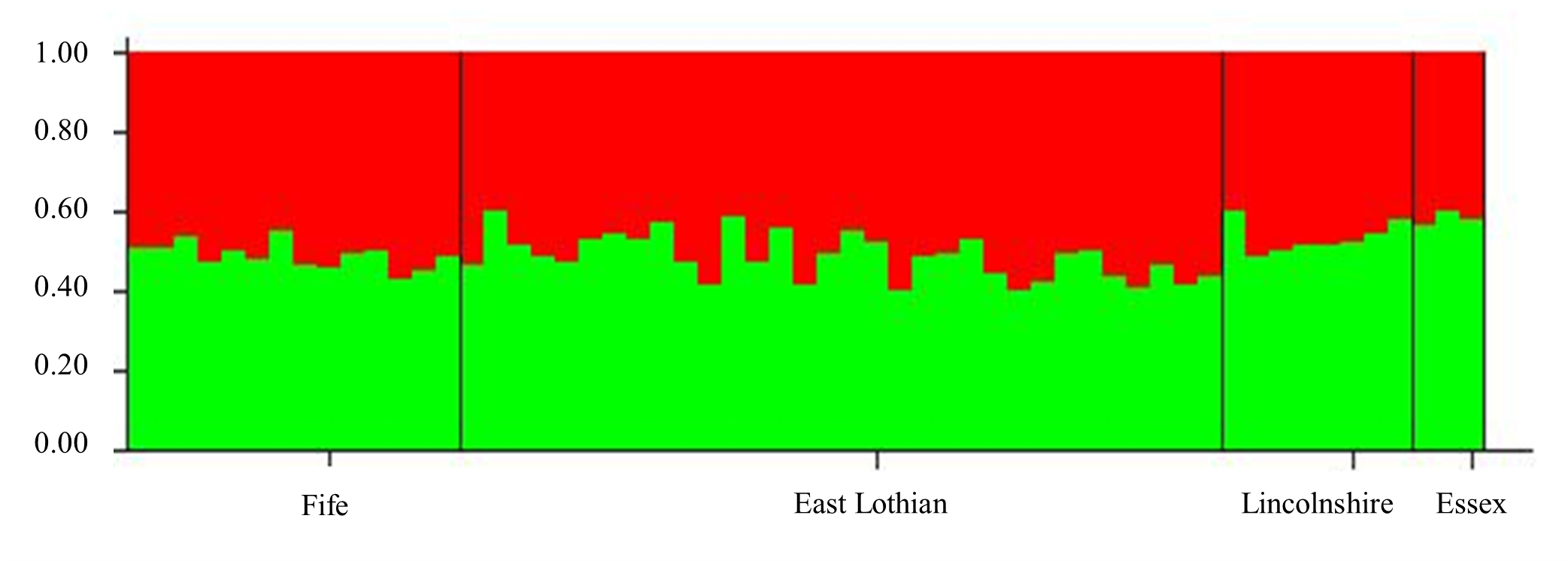

Supplement: Supplementary file 1 — Figure S1. A genetic structure plot of the wild‐caught UK individuals using data from 8 microsatellites. Each bar represents an individual bird and the proportion of each color represents the proportion of different ancestry. The plot shows that all the birds have very similar ancestry. The sampling locations are those shown on the map in Figure 1. [file ECE3-15-e71122-s002.png]

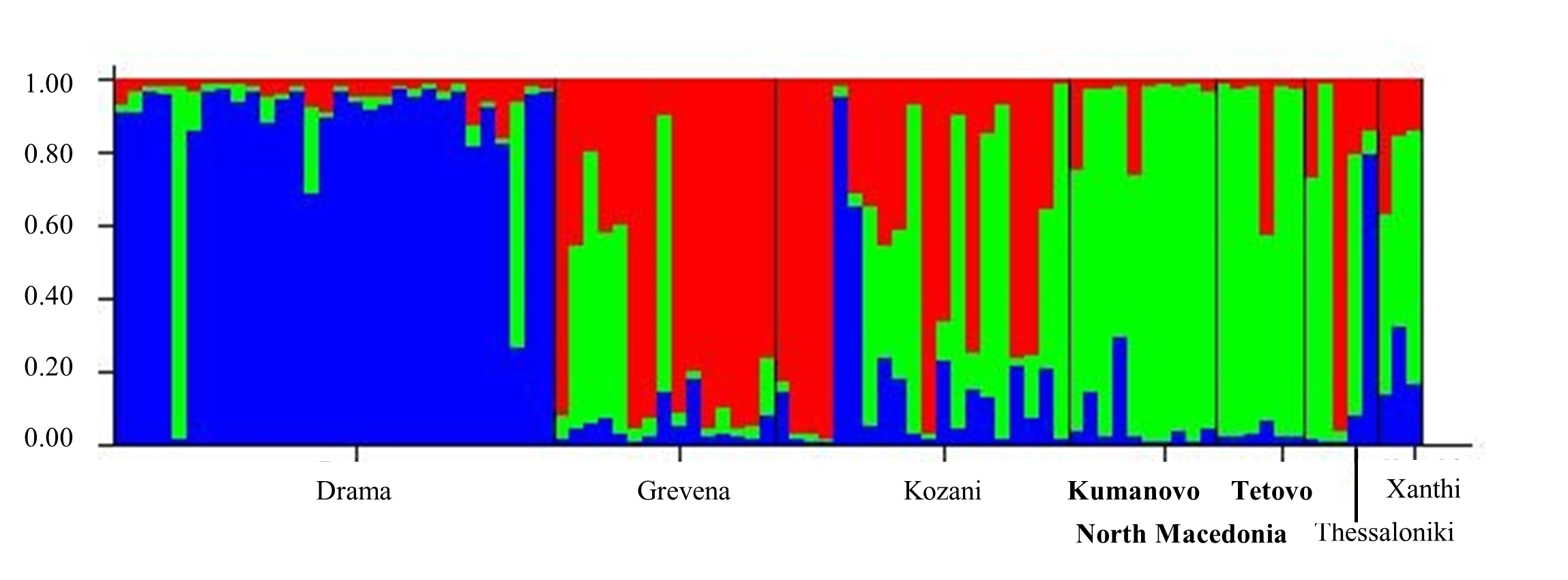

Supplement: Supplementary file 2 — Figure S2. A genetic structure plot of the wild‐caught Greek and North Macedonia individuals using the data from eight microsatellites. Each bar represents an individual bird and the proportion of each color represents the proportion of the different genetic ancestry. Three population clusters were present. The sampling locations are those shown on the map in Figure 1. [file ECE3-15-e71122-s003.png]

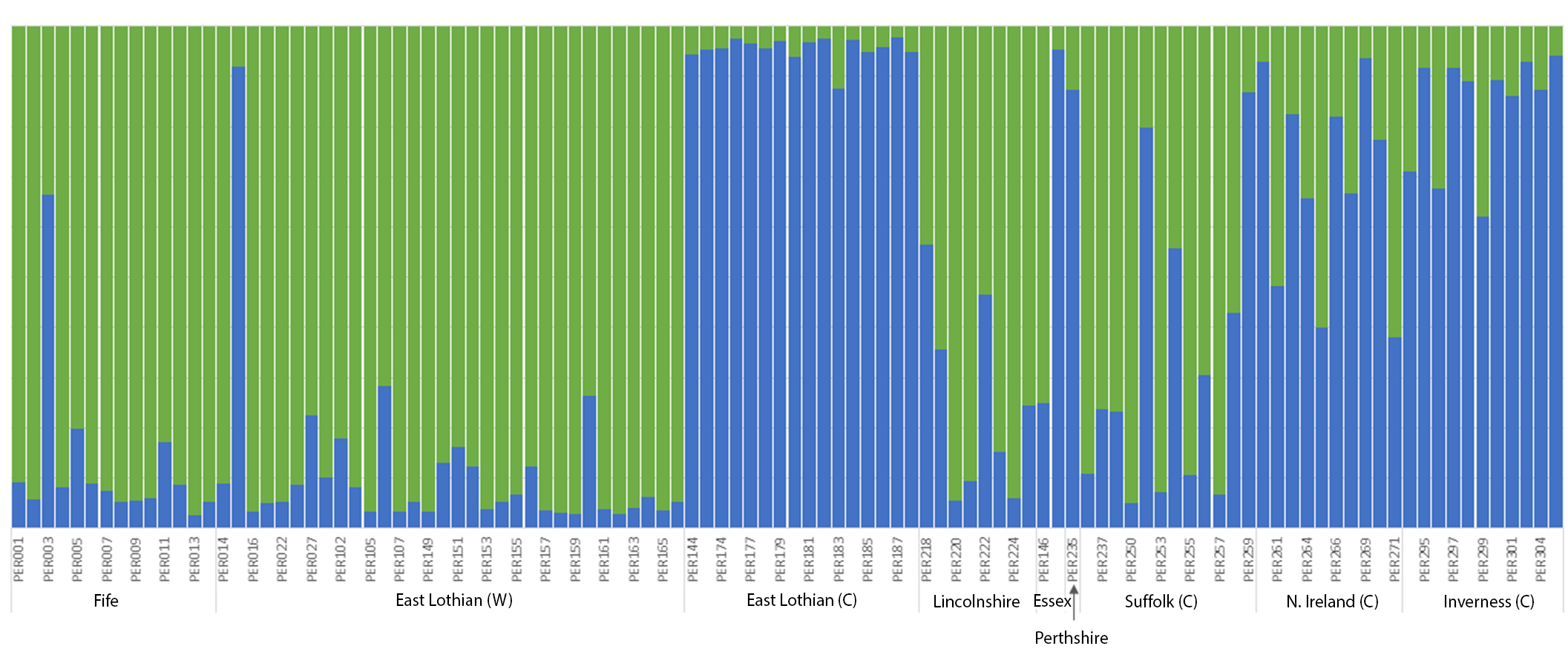

Supplement: Supplementary file 3 — Figure S3. Genetic Structure plot of all birds sampled within the UK. Each bar represents an individual bird and the proportion of each color represents the proportion of the two genetic ancestries. There is very little differentiation with only two clusters. The one incorporates the Scottish and Northern Irish captive (C) individuals (blue) while the other, most of the wild‐caught individuals, along with the captive Suffolk population (green). [file ECE3-15-e71122-s004.png]
