# Supplementary material for: Grey Partridge (Perdix perdix) Introductions: Genetic Survey on Wild and Captive Populations at the Edges of the Range
Source: Ecol Evol. 2025 Mar 21;15(3):e71122. doi: 10.1002/ece3.71122 (PMC11926436; doi:10.1002/ece3.71122)
Supplement: Supplementary file 4 — Data S1 Table S1. Sampling information of all analyzed samples: sample id, CR haplotype for each sample and Accession number, Cyt‐b haplotype for each sample and Accession number, number of microsatellite loci analyzed per sample, samples were ddRAD sequences were obtained, wild or captive, country of origin, sampling region, type of biological material and sex of all analyzed samples. Table S2. Primer information and primer concentration of the eight microsatellite loci used in the two multiplex PCR reactions. Table S3. PCR amplification conditions of multiplex PCR reaction for microsatellite analysis Table S4. Microsatellite loci analyzed. Number of alleles (A), allelic size range in base pairs (R), expected and observed heterozygosity (He, Ho), probability value for Hardy–Weinberg tests (PHW), polymorphic information content (PIC), null alleles per locus (F), sample size (N), mean number of alleles (Amean), and allelic richness (AR) for all samples. Table S5. Allele and genotype frequencies for the top‐scored 15 SNPs. [file ECE3-15-e71122-s001.docx]

**APPENDIX**

**Table S1:** Sampling information of all analyzed samples: sample id, CR haplotype for each sample and Accession number, Cyt-b haplotype for each sample and Accession number, number of microsatellite loci analysed per sample, samples were ddRAD sequences were obtained, wild or captive, country of origin, sampling region, type of biological material and sex of all analysed samples.

|  | **Sample ID** | **CR** | | **Cyt-B** | | **No Msat loci** | **ddRAD** | **Wild/ Captive** | **Country** | **Region** | **Type of sample** | **Sex** |
| --- | --- | --- | --- | --- | --- | --- | --- | --- | --- | --- | --- | --- |
|  |  | **Haplotype ID** | **Accession number GB** | **Haplotype ID** | **Accession number GB** |  | **Accession number GB** |  |  |  |  |  |
| 1 | PER001 | W1 | PQ431691 | CBW3 | PQ295669 | 8 |  | Wild | Scotland | Fife | Feather | Male |
| 2 | PER002 | W1 | PQ431692 | CBW1 | PQ295670 | 8 |  | Wild | Scotland | Fife | Feather | Male |
| 3 | PER003 | W1 | PQ431693 | CBW1 | PQ295671 | 8 |  | Wild | Scotland | Fife | Feather | Male |
| 4 | PER004 |  |  | CBW1 | PQ295672 | 8 |  | Wild | Scotland | Fife | Feather | Female |
| 5 | PER005 | W1 | PQ431694 | CBW1 | PQ295673 | 8 |  | Wild | Scotland | Fife | Feather | Female |
| 6 | PER006 | W1 | PQ431695 | CBW1 | PQ295674 | 8 | SAMN44308948 | Wild | Scotland | Fife | Feather | Female |
| 7 | PER007 | W1 | PQ431696 |  |  | 8 |  | Wild | Scotland | Fife | Feather | Female |
| 8 | PER008 | W1 | PQ431697 |  |  | 8 |  | Wild | Scotland | Fife | Feather | Female |
| 9 | PER009.1 | W1 | PQ431698 | CBW1 | PQ295675 | 8 |  | Wild | Scotland | Fife | Feather | Female |
| 10 | PER010.1 | W1 | PQ431699 | CBW1 | PQ295676 | 8 |  | Wild | Scotland | Fife | Feather | Female |
| 11 | PER011.1 | W1 | PQ431700 | CBW1 | PQ295677 | 8 |  | Wild | Scotland | Fife | Feather | Female |
| 12 | PER012.1 | W1 | PQ431701 | CBW1 | PQ295678 | 8 |  | Wild | Scotland | Fife | Feather | Female |
| 13 | PER013.1 |  |  | CBW1 | PQ295679 | 8 |  | Wild | Scotland | Fife | Feather | Female |
| 14 | PER014.1 | W34 | PQ431702 | CBW1 | PQ295680 | 8 |  | Wild | Scotland | East Lothian | Feather | Female |
| 15 | PER015 | W31 | PQ431703 | CBW1 | PQ295681 | 8 |  | Wild | Scotland | East Lothian | Feather | Male |
| 16 | PER016 | W1 | PQ431704 | CBW1 | PQ295682 | 8 |  | Wild | Scotland | East Lothian | Feather | Female |
| 17 | PER017 | W36 | PQ431705 | CBW1 | PQ295683 |  |  | Wild | Scotland | East Lothian | Feather | Female |
| 18 | PER018 |  |  | CBW2 | PQ295684 | 8 |  | Wild | Scotland | East Lothian | Feather | Female |
| 19 | PER019 | W1 | PQ431706 | CBW1 | PQ295685 |  |  | Wild | Scotland | Fife | Feather | Female |
| 20 | PER021 | W1 | PQ431707 | CBW1 | PQ295686 | 8 |  | Wild | Scotland | Fife | Feather | Female |
| 21 | PER022 | W17 | PQ431708 | CBW1 | PQ295687 | 8 |  | Wild | Scotland | East Lothian | Feather |  |
| 22 | PER023 |  |  | CBW1 | PQ295688 |  |  | Wild | Scotland | Fife | Feather |  |
| 23 | PER024 | W17 | PQ431709 |  |  | 8 |  | Wild | Scotland | East Lothian | Feather |  |
| 24 | PER026 | W1 | PQ431710 |  |  |  |  | Wild | Scotland | East Lothian | Feather |  |
| 25 | PER027.1 |  |  |  |  | 8 | SAMN44308949 | Wild | Scotland | East Lothian | Tissue | Female |
| 26 | PER030.1 | W1 | PQ431621 | CBW2 | PQ295602 | 7 | SAMN44308950 | Captive | Greece |  | Blood | Male |
| 27 | PER031.1 | W1 | PQ431622 | CBW2 | PQ295603 | 8 | SAMN44308951 | Captive | Greece |  | Blood | Female |
| 28 | PER037.1 | W1 | PQ431623 | CBW2 | PQ295604 |  | SAMN44308952 | Offspring | Greece |  | Blood | Male |
| 29 | PER041.1 | W1 | PQ431624 | CBW2 | PQ295605 |  | SAMN44308953 | Offspring | Greece |  | Blood | Male |
| 30 | PER042.1 | W1 | PQ431625 | CBW2 | PQ295606 | 8 | SAMN44308954 | Captive | Greece |  | Blood | Male |
| 31 | PER043.1 | W1 | PQ431626 | CBW2 | PQ295607 | 8 | SAMN44308955 | Captive | Greece |  | Blood | Female |
| 32 | PER044.1 |  |  |  |  |  | SAMN44308956 | Offspring | Greece |  | Blood | Female |
| 33 | PER049.1 |  |  |  |  |  | SAMN44308957 | Offspring | Greece |  | Blood | Female |
| 34 | PER050.1 | W1 | PQ431627 | CBW2 | PQ295608 |  |  | Captive | Greece |  | Blood | Male |
| 35 | PER052.1 | W1 | PQ431628 | CBW2 | PQ295609 | 8 | SAMN44308958 | Captive | Greece |  | Blood | Male |
| 36 | PER053.1 | W1 | PQ431629 | CBW2 | PQ295610 | 8 | SAMN44308959 | Captive | Greece |  | Blood | Female |
| 37 | PER054.1 | W1 | PQ431630 | CBW2 | PQ295611 |  | SAMN44308960 | Offspring | Greece |  | Blood | Male |
| 38 | PER061.1 |  |  |  |  |  | SAMN44308961 | Offspring | Greece |  | Blood | Female |
| 39 | PER063.1 | W1 | PQ431631 | CBW2 | PQ295612 | 8 | SAMN44308962 | Captive | Greece |  | Blood | Male |
| 40 | PER064.1 | W1 | PQ431632 | CBW2 | PQ295613 | 8 | SAMN44308963 | Captive | Greece |  | Blood | Female |
| 41 | PER065.1 | W1 | PQ431633 | CBW2 | PQ295614 |  |  | Captive | Greece |  | Blood | Male |
| 42 | PER073 | W1 | PQ431634 | CBW2 | PQ295615 | 8 |  | Captive | Greece |  | Tissue | Female |
| 43 | PER074 | W1 | PQ431635 | CBW2 | PQ295616 | 8 |  | Captive | Greece |  | Tissue | Male |
| 44 | PER075 | W1 | PQ431636 | CBW2 | PQ295617 | 8 |  | Captive | Greece |  | Tissue | Male |
| 45 | PER076.1 | E1 | PQ431683 | CBE1 | PQ295662 | 8 | SAMN44308964 | Wild | Greece | Thessaloniki | Blood | Male |
| 46 | PER077.1 | E1 | PQ431684 | CBE1 | PQ295663 | 8 | SAMN44308965 | Wild | Greece | Thessaloniki | Blood | Male |
| 47 | PER078.1 | E1 | PQ431685 | CBE1 | PQ295664 | 8 | SAMN44308966 | Wild | Greece | Thessaloniki | Blood | Male |
| 48 | PER079.1 | E1 | PQ431686 | CBE1 | PQ295665 | 7 | SAMN44308967 | Wild | Greece | Thessaloniki | Blood | Male |
| 49 | PER080 |  |  | CBE1 | PQ295689 |  |  | Wild | Finland (send) |  | DNA |  |
| 50 | PER081 |  |  | CBE1 | PQ295690 |  |  | Wild | Finland (send) |  | DNA |  |
| 51 | PER083 | E3 | PQ431711 | CBE1 | PQ295691 |  |  | Wild | Finland (send) |  | DNA |  |
| 52 | PER085 | E16 | PQ431712 | CBE1 | PQ295692 |  |  | Wild | Finland (send) |  | DNA |  |
| 53 | PER086 |  |  | CBE1 | PQ295693 |  |  | Wild | Finland (send) |  | DNA |  |
| 54 | PER087 |  |  | CBE1 | PQ295694 |  |  | Wild | Finland (send) |  | DNA |  |
| 55 | PER088 |  |  | CBE1 | PQ295695 |  |  | Wild | Finland (send) |  | DNA |  |
| 56 | PER090 |  |  | CBE1 | PQ295696 |  |  | Wild | Finland (send) |  | DNA |  |
| 57 | PER091 |  |  | CBE1 | PQ295697 |  |  | Wild | Finland (send) |  | DNA |  |
| 58 | PER092 |  |  | CBE1 | PQ295698 |  |  | Wild | Finland (send) |  | DNA |  |
| 59 | PER094 |  |  | CBE1 | PQ295699 |  |  | Wild | Finland (send) |  | DNA |  |
| 60 | PER095 | E17 | PQ431713 | CBE1 | PQ295700 |  |  | Wild | Finland (send) |  | DNA |  |
| 61 | PER096 | E1 | PQ431714 | CBE1 | PQ295701 | 8 |  | Wild | Greece (1999) |  | Tissue |  |
| 62 | PER097 | E1 | PQ431715 | CBE1 | PQ295702 | 8 |  | Wild | Greece (1999) |  | Tissue |  |
| 63 | PER098 | E1 | PQ431716 | CBE1 | PQ295703 | 8 |  | Wild | Greece (1999) |  | Tissue |  |
| 64 | PER099 | E1 | PQ431717 | CBE1 | PQ295704 | 8 | SAMN44308968 | Wild | Greece (1999) |  | Tissue |  |
| 65 | PER100 | E1 | PQ431718 | CBE1 | PQ295705 | 8 | SAMN44308969 | Wild | Greece (1999) |  | Tissue |  |
| 66 | PER101 | W33 | PQ431719 | CBW2 | PQ295706 | 8 | SAMN44308970 | Wild | Scotland | East Lothian | Tissue |  |
| 67 | PER102 | W38 | PQ431720 | CBW2 | PQ295707 | 8 | SAMN44308971 | Wild | Scotland | East Lothian | Tissue |  |
| 68 | PER104 |  |  | CBW1 | PQ295708 | 8 | SAMN44308972 | Wild | Scotland | East Lothian | Tissue |  |
| 69 | PER105 | W17 | PQ431721 | CBW1 | PQ295709 | 8 | SAMN44308973 | Wild | Scotland | East Lothian | Tissue |  |
| 70 | PER106 | W37 | PQ431722 | CBW1 | PQ295710 | 8 |  | Wild | Scotland | East Lothian | Tissue |  |
| 71 | PER107 | W1 | PQ431723 | CBW2 | PQ295711 | 8 | SAMN44308974 | Wild | Scotland | East Lothian | Tissue |  |
| 72 | PER109.1 |  |  |  |  | 8 |  | Wild | Greece | Kozani | Blood | Female |
| 73 | PER110.1 | E1 | PQ431650 | CBE1 | PQ295631 | 8 |  | Wild | Greece | Grevena | Feather | Male |
| 74 | PER111 |  |  |  |  | 7 |  | Wild | Greece | Grevena | Droppings |  |
| 75 | PER112 |  |  |  |  | 7 |  | Wild | Greece | Grevena | Droppings |  |
| 76 | PER113.1 | E20 | PQ431651 | CBE1 | PQ295632 | 8 |  | Wild | Greece | Grevena | Blood | Male |
| 77 | PER114.1 | E1 | PQ431652 | CBE1 | PQ295633 | 8 | SAMN44308975 | Wild | Greece | Grevena | Blood | Male |
| 78 | PER115.1 | E20 | PQ431653 | CBE1 | PQ295634 | 8 | SAMN44308976 | Wild | Greece | Grevena | Blood | Male |
| 79 | PER116 | E1 | PQ431654 | CBE1 | PQ295635 | 8 |  | Wild | Greece | Grevena | Tissue |  |
| 80 | PER117.1 | E1 | PQ431687 | CBE1 | PQ295666 | 8 | SAMN44308977 | Wild | Greece | Thessaloniki | Tissue |  |
| 81 | PER118 | E22 | PQ431663 | CBE1 | PQ295644 | 8 | SAMN44308978 | Wild | Greece | Kozani | Feather | Female |
| 82 | PER119 | E22 | PQ431664 | CBE1 | PQ295645 | 8 | SAMN44308979 | Wild | Greece | Kozani | Feather | Male |
| 83 | PER120 | E22 | PQ431665 |  |  | 8 | SAMN44308980 | Wild | Greece | Kozani | Feather | Male |
| 84 | PER121 | E1 | PQ431666 | CBE1 | PQ295646 | 8 | SAMN44308981 | Wild | Greece | Kozani | Feather | Female |
| 85 | PER122 | E22 | PQ431667 | CBE2 | PQ295647 | 8 | SAMN44308982 | Wild | Greece | Kozani | Feather | Female |
| 86 | PER123 | E1 | PQ431668 | CBE1 | PQ295648 | 8 | SAMN44308983 | Wild | Greece | Kozani | Feather | Female |
| 87 | PER124 | E23 | PQ431669 | CBE1 | PQ295649 | 8 | SAMN44308984 | Wild | Greece | Kozani | Feather | Male |
| 88 | PER125 | E24 | PQ431670 | CBE1 | PQ295650 | 8 | SAMN44308985 | Wild | Greece | Kozani | Feather | Male |
| 89 | PER126 | E22 | PQ431671 | CBE1 | PQ295651 | 8 | SAMN44308986 | Wild | Greece | Kozani | Feather | Male |
| 90 | PER127.1 | E22 | PQ431672 | CBE1 | PQ295652 | 8 | SAMN44308987 | Wild | Greece | Kozani | Feather | Male |
| 91 | PER128.1 | E22 | PQ431673 | CBE2 | PQ295653 | 8 |  | Wild | Greece | Kozani | Feather | Male |
| 92 | PER129.1 | E22 | PQ431674 | CBE1 | PQ295654 | 8 |  | Wild | Greece | Kozani | Feather | Male |
| 93 | PER130 | E1 | PQ431655 | CBE1 | PQ295636 | 6 | SAMN44308988 | Wild | Greece | Grevena | Feather | Female |
| 94 | PER131 | E22 | PQ431656 | CBE2 | PQ295637 | 8 |  | Wild | Greece | Grevena | Feather | Male |
| 95 | PER132 | E20 | PQ431657 | CBE1 | PQ295638 | 8 | SAMN44308989 | Wild | Greece | Grevena | Feather | Male |
| 96 | PER133 | E20 | PQ431658 | CBE1 | PQ295639 | 8 | SAMN44308990 | Wild | Greece | Grevena | Feather | Male |
| 97 | PER134 | E20 | PQ431659 | CBE1 | PQ295640 | 8 | SAMN44308991 | Wild | Greece | Grevena | Feather | Male |
| 98 | PER135 | E20 | PQ431660 | CBE1 | PQ295641 | 8 | SAMN44308992 | Wild | Greece | Grevena | Feather | Male |
| 99 | PER136 | E1 | PQ431591 | CBE1 | PQ295572 | 8 | SAMN44308993 | Wild | Greece | Drama | Feather | Male |
| 100 | PER137 | W1 | PQ431643 | CBW1 | PQ295624 | 8 | SAMN44308994 | Captive | Greece |  | Feather | Male |
| 101 | PER138 | W1 | PQ431644 | CBW1 | PQ295625 | 8 | SAMN44308995 | Captive | Greece |  | Feather | Female |
| 102 | PER139 | W17 | PQ431645 | CBW1 | PQ295626 | 8 | SAMN44308996 | Captive | Greece |  | Feather | Male |
| 103 | PER140 | W17 | PQ431646 | CBW1 | PQ295627 | 8 |  | Captive | Greece |  | Feather | Male |
| 104 | PER141 | W1 | PQ431647 | CBW1 | PQ295628 | 6 | SAMN44308997 | Captive | Greece |  | Feather | Male |
| 105 | PER142 | W17 | PQ431648 | CBW1 | PQ295629 | 8 | SAMN44308998 | Captive | Greece |  | Feather | Male |
| 106 | PER143 | W1 | PQ431649 | CBW1 | PQ295630 | 8 | SAMN44308999 | Captive | Greece |  | Feather | Male |
| 107 | PER144 | W1 | PQ431724 | CBW2 | PQ295712 | 8 |  | Captive | Scotland | South Lanarkshire | Feather | Male |
| 108 | PER145 |  |  | CBW2 | PQ295713 | 8 |  | Captive | Scotland | South Lanarkshire | Feather | Male |
| 109 | PER146 | W1 | PQ431725 | CBW1 | PQ295714 | 8 |  | Wild | England | Essex | DNA | Male |
| 110 | PER147 | W1 | PQ431726 |  |  | 8 | SAMN44309000 | Wild | England | Essex | DNA | Female |
| 111 | PER148 | W1 | PQ431727 | CBW2 | PQ295715 | 8 |  | Wild | Scotland | East Lothian | Feather |  |
| 112 | PER149 | W1 | PQ431728 | CBW2 | PQ295716 | 8 |  | Wild | Scotland | East Lothian | Feather |  |
| 113 | PER150 | W17 | PQ431729 | CBW1 | PQ295717 | 8 |  | Wild | Scotland | East Lothian | Feather |  |
| 114 | PER151 | W1 | PQ431730 | CBW2 | PQ295718 | 8 |  | Wild | Scotland | East Lothian | Feather |  |
| 115 | PER152 | W1 | PQ431731 | CBW1 | PQ295719 | 8 |  | Wild | Scotland | East Lothian | Feather |  |
| 116 | PER153 |  |  | CBW1 | PQ295720 | 8 |  | Wild | Scotland | East Lothian | Feather |  |
| 117 | PER154 | W1 | PQ431732 | CBW2 | PQ295721 | 8 |  | Wild | Scotland | East Lothian | Feather |  |
| 118 | PER155 | W4 | PQ431733 | CBW1 | PQ295722 | 8 |  | Wild | Scotland | East Lothian | Feather |  |
| 119 | PER156 | W1 | PQ431734 | CBW1 | PQ295723 | 8 |  | Wild | Scotland | East Lothian | Feather |  |
| 120 | PER157 | W4 | PQ431735 | CBW1 | PQ295724 | 8 |  | Wild | Scotland | East Lothian | Feather |  |
| 121 | PER158 | W17 | PQ431736 | CBW1 | PQ295725 | 8 |  | Wild | Scotland | East Lothian | Feather |  |
| 122 | PER159 | W4 | PQ431737 |  |  | 8 |  | Wild | Scotland | East Lothian | Feather |  |
| 123 | PER160 | W4 | PQ431738 | CBW1 | PQ295726 | 8 |  | Wild | Scotland | East Lothian | Feather |  |
| 124 | PER161 |  |  | CBW2 | PQ295727 | 8 |  | Wild | Scotland | East Lothian | Feather |  |
| 125 | PER162 | W17 | PQ431739 |  |  | 8 |  | Wild | Scotland | East Lothian | Feather |  |
| 126 | PER163 | W4 | PQ431740 | CBW1 | PQ295728 | 8 |  | Wild | Scotland | East Lothian | Feather |  |
| 127 | PER164 | W17 | PQ431741 | CBW1 | PQ295729 | 8 |  | Wild | Scotland | East Lothian | Feather |  |
| 128 | PER165 | W4 | PQ431742 | CBW1 | PQ295730 | 8 |  | Wild | Scotland | East Lothian | Feather |  |
| 129 | PER166 | W4 | PQ431743 | CBW1 | PQ295731 | 8 |  | Wild | Scotland | East Lothian | Feather |  |
| 130 | PER168.1 | W1 | PQ431637 | CBW2 | PQ295618 | 8 | SAMN44309001 | Hybrids | Greece |  | Blood | Male |
| 131 | PER169.1 | W1 | PQ431638 | CBW2 | PQ295619 | 8 | SAMN44309002 | Hybrids | Greece |  | Blood | Female |
| 132 | PER170.1 | W1 | PQ431639 | CBW2 | PQ295620 | 8 | SAMN44309003 | Hybrids | Greece |  | Blood | Male |
| 133 | PER171.1 | W1 | PQ431640 | CBW2 | PQ295621 | 6 |  | Hybrids | Greece |  | Blood | Female |
| 134 | PER172.1 | W1 | PQ431641 | CBW2 | PQ295622 | 8 | SAMN44309004 | Hybrids | Greece |  | Blood | Male |
| 135 | PER173.1 | W1 | PQ431642 | CBW2 | PQ295623 | 8 | SAMN44309005 | Hybrids | Greece |  | Blood | Female |
| 136 | PER174 |  |  |  |  | 8 |  | Captive | Scotland | East Lothian | Blood | Male |
| 137 | PER175.1 | W1 | PQ431744 | CBW2 | PQ295732 | 8 |  | Captive | Scotland | East Lothian | Blood | Male |
| 138 | PER177.1 | W1 | PQ431745 |  |  | 8 |  | Captive | Scotland | East Lothian | Blood | Female |
| 139 | PER178.1 | W1 | PQ431746 | CBW2 | PQ295733 | 8 |  | Captive | Scotland | East Lothian | Blood | Female |
| 140 | PER179 | W1 | PQ431747 | CBW2 | PQ295734 | 8 |  | Captive | Scotland | East Lothian | Feather | Male |
| 141 | PER180 |  |  | CBW2 | PQ295735 | 8 |  | Captive | Scotland | East Lothian | Feather | Male |
| 142 | PER181 | W1 | PQ431748 | CBW1 | PQ295736 | 8 |  | Captive | Scotland | East Lothian | Feather | Male |
| 143 | PER182 | W1 | PQ431749 | CBW2 | PQ295737 | 8 |  | Captive | Scotland | East Lothian | Feather | Male |
| 144 | PER183 | W1 | PQ431750 | CBW2 | PQ295738 | 8 |  | Captive | Scotland | East Lothian | Feather | Male |
| 145 | PER184 | W1 | PQ431751 | CBW2 | PQ295739 | 8 |  | Captive | Scotland | East Lothian | Feather | Male |
| 146 | PER185 | W1 | PQ431752 | CBW1 | PQ295740 | 8 | SAMN44309006 | Captive | Scotland | East Lothian | Feather | Female |
| 147 | PER186 | W1 | PQ431753 |  |  | 8 | SAMN44309007 | Captive | Scotland | East Lothian | Feather | Female |
| 148 | PER187 |  |  | CBW2 | PQ295741 | 8 |  | Captive | Scotland | East Lothian | Feather | Female |
| 149 | PER188 | W1 | PQ431754 | CBW1 | PQ295742 | 8 | SAMN44309008 | Captive | Scotland | East Lothian | Feather | Female |
| 150 | PER189.1 | E1 | PQ431592 | CBE1 | PQ295573 | 8 |  | Wild | Greece | Drama | Blood | Male |
| 151 | PER190.1 | E1 | PQ431593 | CBE1 | PQ295574 | 8 |  | Wild | Greece | Drama | Blood | Male |
| 152 | PER191 | E1 | PQ431594 | CBE1 | PQ295575 | 8 |  | Wild | Greece | Drama | Feather | Male |
| 153 | PER192 | E1 | PQ431595 | CBE1 | PQ295576 | 8 |  | Wild | Greece | Drama | Feather | Male |
| 154 | PER193 | E1 | PQ431596 | CBE1 | PQ295577 | 8 | SAMN44309009 | Wild | Greece | Drama | Feather | Male |
| 155 | PER194 | E1 | PQ431597 | CBE1 | PQ295578 | 8 | SAMN44309010 | Wild | Greece | Drama | Feather | Male |
| 156 | PER195 | E1 | PQ431598 | CBE1 | PQ295579 | 8 | SAMN44309011 | Wild | Greece | Drama | Feather | Male |
| 157 | PER196 | E1 | PQ431599 | CBE1 | PQ295580 | 8 |  | Wild | Greece | Drama | Feather | Male |
| 158 | PER197 | E1 | PQ431600 | CBE1 | PQ295581 | 8 | SAMN44309012 | Wild | Greece | Drama | Feather | Male |
| 159 | PER198 | E1 | PQ431601 | CBE1 | PQ295582 | 8 | SAMN44309013 | Wild | Greece | Drama | Feather | Male |
| 160 | PER199 | E1 | PQ431602 | CBE1 | PQ295583 | 8 |  | Wild | Greece | Drama | Feather | Male |
| 161 | PER200 | E1 | PQ431603 | CBE1 | PQ295584 | 8 | SAMN44309014 | Wild | Greece | Drama | Feather | Male |
| 162 | PER201 | E1 | PQ431604 | CBE1 | PQ295585 | 8 | SAMN44309015 | Wild | Greece | Drama | Feather | Male |
| 163 | PER202.1 | E1 | PQ431605 | CBE1 | PQ295586 | 8 |  | Wild | Greece | Drama | Feather | Male |
| 164 | PER203.1 | E1 | PQ431606 | CBE1 | PQ295587 | 8 |  | Wild | Greece | Drama | Feather | Male |
| 165 | PER204.1 | E1 | PQ431607 | CBE1 | PQ295588 | 7 |  | Wild | Greece | Drama | Feather | Male |
| 166 | PER205 | E1 | PQ431608 | CBE1 | PQ295589 | 7 |  | Wild | Greece | Drama | Feather | Male |
| 167 | PER206 | E1 | PQ431609 | CBE1 | PQ295590 | 7 |  | Wild | Greece | Drama | Feather | Male |
| 168 | PER207 | E1 | PQ431610 | CBE1 | PQ295591 | 8 |  | Wild | Greece | Drama | Feather | Male |
| 169 | PER208 | E1 | PQ431611 | CBE1 | PQ295592 | 8 |  | Wild | Greece | Drama | Feather | Male |
| 170 | PER209.1 | E1 | PQ431612 | CBE1 | PQ295593 | 8 |  | Wild | Greece | Drama | Feather | Male |
| 171 | PER210.1 | E1 | PQ431613 | CBE1 | PQ295594 | 8 |  | Wild | Greece | Drama | Feather | Male |
| 172 | PER211 | E1 | PQ431675 | CBE1 | PQ295655 | 8 | SAMN44309016 | Wild | Greece | Kozani | Feather | Male |
| 173 | PER212 | E1 | PQ431676 | CBE1 | PQ295656 | 8 |  | Wild | Greece | Kozani | Feather | Male |
| 174 | PER213 | E1 | PQ431677 | CBE1 | PQ295657 | 8 |  | Wild | Greece | Kozani | Feather | Male |
| 175 | PER214 | E1 | PQ431678 | CBE1 | PQ295658 | 8 | SAMN44309017 | Wild | Greece | Kozani | Feather | Male |
| 176 | PER215 | E18 | PQ431679 |  |  | 8 | SAMN44309018 | Wild | Greece | Kozani | Feather | Female |
| 177 | PER216 | E22 | PQ431680 | CBE1 | PQ295659 | 8 |  | Wild | Greece | Kozani | Feather | Female |
| 178 | PER217 | E22 | PQ431681 | CBE2 | PQ295660 | 8 |  | Wild | Greece | Kozani | Feather | Male |
| 179 | PER218 | W1 | PQ431755 | CBW1 | PQ295743 | 8 | SAMN44309019 | Wild | England | Lincolnshire | Tissue | Female |
| 180 | PER219 | W1 | PQ431756 | CBW1 | PQ295744 | 8 | SAMN44309020 | Wild | England | Lincolnshire | Tissue | Male |
| 181 | PER220 | W1 | PQ431757 | CBW1 | PQ295745 | 8 | SAMN44309021 | Wild | England | Lincolnshire | Tissue | Female |
| 182 | PER221 | W1 | PQ431758 |  |  | 8 | SAMN44309022 | Wild | England | Lincolnshire | Tissue | Female |
| 183 | PER222 | W1 | PQ431759 |  |  | 8 | SAMN44309023 | Wild | England | Lincolnshire | Tissue | Female |
| 184 | PER223 | W1 | PQ431760 |  |  | 8 | SAMN44309024 | Wild | England | Lincolnshire | Tissue | Male |
| 185 | PER224 | W1 | PQ431761 |  |  | 8 | SAMN44309025 | Wild | England | Lincolnshire | Tissue | Female |
| 186 | PER225 | W1 | PQ431762 | CBW1 | PQ295746 | 8 | SAMN44309026 | Wild | England | Lincolnshire | Tissue | Male |
| 187 | PER226 | E20 | PQ431661 | CBE1 | PQ295642 | 7 |  | Wild | Greece | Grevena | Feather | Female |
| 188 | PER227 | E1 | PQ431662 | CBE1 | PQ295643 | 8 | SAMN44309027 | Wild | Greece | Grevena | Feather | Male |
| 189 | PER228 | E1 | PQ431614 | CBE1 | PQ295595 | 8 |  | Wild | Greece | Drama | Feather | Male |
| 190 | PER229 | E1 | PQ431615 | CBE1 | PQ295596 | 8 |  | Wild | Greece | Drama | Feather | Male |
| 191 | PER230 | E1 | PQ431616 | CBE1 | PQ295597 | 8 |  | Wild | Greece | Drama | Feather | Male |
| 192 | PER231 | E1 | PQ431617 | CBE1 | PQ295598 | 8 |  | Wild | Greece | Drama | Feather | Male |
| 193 | PER232 | E1 | PQ431618 | CBE1 | PQ295599 | 7 |  | Wild | Greece | Drama | Feather | Male |
| 194 | PER233 | E1 | PQ431619 | CBE1 | PQ295600 | 8 |  | Wild | Greece | Drama | Feather | Male |
| 195 | PER234 | E1 | PQ431620 | CBE1 | PQ295601 | 8 |  | Wild | Greece | Drama | Feather | Male |
| 196 | PER235 | W1 | PQ431763 | CBW2 | PQ295747 | 8 |  | Wild | Scotland | Perthshire | Tissue |  |
| 197 | PER236 | W1 | PQ431764 | CBW1 | PQ295748 | 7 |  | Captive | UK | Suffolk | Egg |  |
| 198 | PER237 | W1 | PQ431765 | CBW1 | PQ295749 | 8 |  | Captive | UK | Suffolk | Egg |  |
| 199 | PER238 | W4 | PQ431766 | CBW1 | PQ295750 |  |  | Captive | UK | Suffolk | Egg |  |
| 200 | PER239 |  |  | CBW1 | PQ295751 |  |  | Captive | UK | Suffolk | Egg |  |
| 201 | PER240 |  |  | CBW1 | PQ295752 |  |  | Captive | UK | Suffolk | Egg |  |
| 202 | PER248 | W4 | PQ431767 | CBW1 | PQ295753 | 6 |  | Captive | UK | Suffolk | Egg |  |
| 203 | PER249 | W1 | PQ431768 |  |  |  |  | Captive | UK | Suffolk | Egg |  |
| 204 | PER250 | W1 | PQ431769 | CBW1 | PQ295754 | 8 |  | Captive | UK | Suffolk | Egg |  |
| 205 | PER252 | W1 | PQ431770 | CBW1 | PQ295755 | 6 |  | Captive | UK | Suffolk | Egg |  |
| 206 | PER253 | W1 | PQ431771 | CBW2 | PQ295756 | 6 |  | Captive | UK | Suffolk | Egg |  |
| 207 | PER254 | W1 | PQ431772 | CBW1 | PQ295757 | 8 |  | Captive | UK | Suffolk | Egg |  |
| 208 | PER255 | W35 | PQ431773 | CBW1 | PQ295758 | 8 |  | Captive | UK | Suffolk | Egg |  |
| 209 | PER256 | W31 | PQ431774 | CBW1 | PQ295759 | 8 |  | Captive | UK | Suffolk | Egg |  |
| 210 | PER257 | W1 | PQ431775 | CBW1 | PQ295760 | 8 |  | Captive | UK | Suffolk | Egg |  |
| 211 | PER258 | W35 | PQ431776 | CBW1 | PQ295761 | 8 |  | Captive | UK | Suffolk | Egg |  |
| 212 | PER259 | W1 | PQ431777 | CBW1 | PQ295762 | 8 |  | Captive | UK | Suffolk | Egg |  |
| 213 | PER260 | W17 | PQ431778 | CBW1 | PQ295763 | 6 |  | Captive | UK | Northern Ireland | Egg |  |
| 214 | PER261 | W4 | PQ431779 | CBW1 | PQ295764 | 8 |  | Captive | UK | Northern Ireland | Egg |  |
| 215 | PER262 | W17 | PQ431780 | CBW1 | PQ295765 | 8 |  | Captive | UK | Northern Ireland | Egg |  |
| 216 | PER263 |  |  | CBW1 | PQ295766 |  |  | Captive | UK | Northern Ireland | Egg |  |
| 217 | PER264 | W17 | PQ431781 | CBW1 | PQ295767 | 8 |  | Captive | UK | Northern Ireland | Egg |  |
| 218 | PER265 |  |  | CBW1 | PQ295768 | 6 |  | Captive | UK | Northern Ireland | Egg |  |
| 219 | PER266 | W17 | PQ431782 | CBW1 | PQ295769 | 8 |  | Captive | UK | Northern Ireland | Egg |  |
| 220 | PER267 | W17 | PQ431783 | CBW1 | PQ295770 | 8 |  | Captive | UK | Northern Ireland | Egg |  |
| 221 | PER268 | W1 | PQ431784 | CBW1 | PQ295771 |  |  | Captive | UK | Northern Ireland | Egg |  |
| 222 | PER269 | W17 | PQ431785 | CBW1 | PQ295772 | 8 |  | Captive | UK | Northern Ireland | Egg |  |
| 223 | PER270 | W17 | PQ431786 | CBW1 | PQ295773 | 8 |  | Captive | UK | Northern Ireland | Egg |  |
| 224 | PER271 | W4 | PQ431787 | CBW1 | PQ295774 | 8 |  | Captive | UK | Northern Ireland | Egg |  |
| 225 | PER272 | E1 | PQ431688 | CBE1 | PQ295667 | 6 |  | Wild | Greece | Xanthi | Feather | Male |
| 226 | PER273 | E1 | PQ431689 | CBE1 | PQ295668 | 6 | SAMN44309028 | Wild | Greece | Xanthi | Feather | Male |
| 227 | PER274 | E1 | PQ431690 |  |  | 6 |  | Wild | Greece | Xanthi | Feather | Male |
| 228 | PER275 | E1 | PQ431682 | CBE1 | PQ295661 | 6 |  | Wild | Greece | Kastoria | Tissue |  |
| 229 | PER276 | E21 | PQ431575 | CBE1 | PQ295559 | 7 | SAMN44309029 | Wild | North Macedonia | Kumanovo | Feather | Male |
| 230 | PER277 | W1 | PQ431576 | CBW1 | PQ295560 | 7 |  | Wild | North Macedonia | Kumanovo | Feather | Male |
| 231 | PER278 | E19 | PQ431577 | CBE1 | PQ295561 | 8 |  | Wild | North Macedonia | Kumanovo | Feather | Male |
| 232 | PER279 | W1 | PQ431578 | CBW1 | PQ295562 | 8 |  | Wild | North Macedonia | Kumanovo | Feather | Male |
| 233 | PER280 | E16 | PQ431579 | CBE1 | PQ295563 | 8 | SAMN44309030 | Wild | North Macedonia | Kumanovo | Feather | Male |
| 234 | PER281 | E16 | PQ431580 | CBE1 | PQ295564 | 8 | SAMN44309031 | Wild | North Macedonia | Kumanovo | Feather | Male |
| 235 | PER282 | E1 | PQ431581 | CBE1 | PQ295565 | 7 | SAMN44309032 | Wild | North Macedonia | Kumanovo | Feather | Male |
| 236 | PER283 | E16 | PQ431582 | CBE1 | PQ295566 | 7 |  | Wild | North Macedonia | Kumanovo | Feather | Male |
| 237 | PER284 | E1 | PQ431583 | CBE1 | PQ295567 | 7 |  | Wild | North Macedonia | Kumanovo | Feather | Male |
| 238 | PER285 | E16 | PQ431584 | CBE1 | PQ295568 | 8 |  | Wild | North Macedonia | Kumanovo | Feather | Female |
| 239 | PER286 | W32 | PQ431585 | CBW1 | PQ295569 | 8 | SAMN44309033 | Wild | North Macedonia | Tetovo | Feather | Male |
| 240 | PER287 | E1 | PQ431586 | CBE1 | PQ295570 | 7 |  | Wild | North Macedonia | Tetovo | Feather | Male |
| 241 | PER288 | W1 | PQ431587 |  |  | 7 |  | Wild | North Macedonia | Tetovo | Tissue | Male |
| 242 | PER289 | E18 | PQ431588 |  |  | 7 |  | Wild | North Macedonia | Tetovo | Tissue | Male |
| 243 | PER290 | W1 | PQ431589 |  |  | 6 |  | Wild | North Macedonia | Tetovo | Tissue | Male |
| 244 | PER291 | E1 | PQ431590 | CBE1 | PQ295571 | 7 | SAMN44309034 | Wild | North Macedonia | Tetovo | Tissue | Male |
| 245 | PER294 | W1 | PQ431788 | CBW2 | PQ295775 | 7 |  | Captive | Scotland | Inverness-shire | Tissue | Male |
| 246 | PER295 |  |  | CBW1 | PQ295776 | 8 |  | Captive | Scotland | Inverness-shire | Egg |  |
| 247 | PER296 | W4 | PQ431789 | CBW1 | PQ295777 | 8 |  | Captive | Scotland | Inverness-shire | Egg |  |
| 248 | PER297 | W1 | PQ431790 | CBW1 | PQ295778 | 8 |  | Captive | Scotland | Inverness-shire | Egg |  |
| 249 | PER298 | W1 | PQ431791 | CBW2 | PQ295779 | 8 |  | Captive | Scotland | Inverness-shire | Egg |  |
| 250 | PER299 | W4 | PQ431792 | CBW1 | PQ295780 | 6 |  | Captive | Scotland | Inverness-shire | Egg |  |
| 251 | PER300 | W4 | PQ431793 | CBW1 | PQ295781 | 8 |  | Captive | Scotland | Inverness-shire | Egg |  |
| 252 | PER301 | W4 | PQ431794 | CBW1 | PQ295782 | 8 |  | Captive | Scotland | Inverness-shire | Egg |  |
| 253 | PER302 | W1 | PQ431795 | CBW2 | PQ295783 |  |  | Captive | Scotland | Inverness-shire | Egg |  |
| 254 | PER303 | W4 | PQ431796 | CBW1 | PQ295784 | 8 |  | Captive | Scotland | Inverness-shire | Egg |  |
| 255 | PER304 | W1 | PQ431797 | CBW1 | PQ295785 | 8 |  | Captive | Scotland | Inverness-shire | Egg |  |
| 256 | PER305 | W1 | PQ431798 | CBW2 | PQ295786 | 8 |  | Captive | Scotland | Inverness-shire | Egg |  |

**Table S2:** Primer information and primer concentration of the 8 microsatellite loci used in the two multiplex PCR reactions.

|  |  | **Primer Sequence** | **Fragment size** | **Final concentration** | **Reference** |
| --- | --- | --- | --- | --- | --- |
| **Panel A** | **MNT412** | F: CCCATGTGAGCAGTGAATTG | 235-273 | 0.33 pmol/μl | (Bech *et al.*, 2010) |
|  |  | R: GTCATCACAGTGGAGGATCG |  |  |  |
|  | **Aru1G4** | F : CTGCAGTCACACAAGGCTAC | 140-166 | 0.16 pmol/μl | (Ferrero *et al.*, 2007) |
|  |  | R: AGTGGGTCAAGGATGAGTGG |  |  |  |
|  | **Aru1A1** | F: GGAAGCCAGATGAACCAAGG | 203-227 | 0.16 pmol/μl | (Ferrero *et al.*, 2007) |
|  |  | R: ATGCATGCGTGGAGGCTGAG |  |  |  |
|  | **MNT45** | F: ACATGGAGGCAGAGAACCTC | 107-113 | 0.33 pmol/μl | (Bech *et al.*, 2010) |
|  |  | R: TGTCAGCCTGAATGTTTCCTC |  |  |  |
| **Panel B** | **MNT12** | F: AGGTGTTTTTGGGCAGTCTC | 140-180 | 0.25 pmol/μl | (Bech *et al.*, 2010) |
|  |  | R: TGCAAGCACCATCTGCTAAG |  |  |  |
|  | **MNT408** | F: GTGTCCCTGCCACACTACAG | 219-237 | 0.33 pmol/μl | (Bech *et al.*, 2010) |
|  |  | R: GGGAATTTGCTCCAACTGAC |  |  |  |
|  | **MNT404** | F: AACCAGCTCTGGAGATACCG | 250-254 | 0.16 pmol/μl | (Bech *et al.*, 2010) |
|  |  | R: GGACTGCAAGGACAACATCC |  |  |  |
|  | **MNT477** | F: TTCACCACGCTCATTCAAAG | 229-263 | 0.25 pmol/μl | (Bech *et al.*, 2010) |
|  |  | R:TCCAAAATGTGACTAGATGATAAAGTG |  |  |  |

**Table S3:** PCR amplification conditions of multiplex PCR reaction for microsatellite analysis

| **Steps** | **Temperature** | **Time** | **Cycles** |
| --- | --- | --- | --- |
| **Initial denaturation** | 95^ο^C | 15min |  |
| **Denaturation** | 94^o^C | 30sec |  |
| **Annealing** | 60^o^C-50^o^C | 45sec | 19 |
| **Extension** | 72^ο^C | 60sec |  |
| **Denaturation** | 94^ο^C | 30sec |  |
| **Annealing** | 50^o^C | 45sec | 20 |
| **Extension** | 72^o^C | 60sec |  |
| **Final Extension** | 72^o^C | 20min |  |
|  |  |  |  |

**Table S4.** Microsatellite loci analysed. Number of alleles (A), allelic size range in base pairs (R), expected and observed heterozygosity (He, Ho), probability value for Hardy-Weinberg tests (P_HW_), polymorphic information content (PIC), null alleles per locus (F), sample size (N), mean number of alleles (Amean) and allelic richness (A_R_) for all samples.

|  | ΜΝΤ412 | Aru1G4 | Aru1A1 | MNT45 | MNT12 | MNT404 | MNT477 | MNT408 |
| --- | --- | --- | --- | --- | --- | --- | --- | --- |
| Α | 18 | 14 | 3 | 4 | 27 | 3 | 12 | 10 |
| R | 235-273 | 140-166 | 203-227 | 107-113 | 140-180 | 250-254 | 229-263 | 227-237 |
| He | 0.893 | 0,704 | 0.394 | 0.474 | 0.886 | 0.338 | 0.701 | 0.721 |
| HO | 0,741 | 0,582 | 0.400 | 0.366 | 0.556 | 0.214 | 0.445 | 0.593 |
| PHW | *** | 0.005 | 0,862 | 0.0062 | *** | *** | *** | 0.005 |
| PIC | 0.882 | 0,666 | 0.359 | 0.3767 | 0.876 | 0.290 | 0.666 | 0.665 |
| F | 0,0942 | 0.0955 | -0.0103 | 0.1283 | 0,233 | 0,219 | 0,228 | 0.099 |

**Table S5.** Allele and genotype frequencies for the top-scored 15 SNPs.

|  | Genotyped | 1st Allele | 2nd Allele | Genotypes | | |
| --- | --- | --- | --- | --- | --- | --- |
| SNP6107_124 |  | freq: 2 | freq: 4 | 22 | 44 | 24 |
| East Wild | 37 | 0.014 | 0.986 | 0 | 0.973 | 0.027 |
| West_Wild | 16 | 0.969 | 0.031 | 0.938 | 0 | 0.062 |
| Total | 53 | 0.302 | 0.698 | 0.283 | 0.679 | 0.038 |
|  |  |  |  |  |  |  |
| SNP14657_117 |  | freq: 2 | freq: 4 | 44 | 22 | 42 |
| East Wild | 38 | 1 | 0 | 0 | 1 | 0 |
| West_Wild | 14 | 0.071 | 0.929 | 0.857 | 0 | 0.143 |
| Total | 52 | 0.75 | 0.25 | 0.231 | 0.731 | 0.038 |
|  |  |  |  |  |  |  |
| SNP3226_123 |  | freq: 2 | freq: 4 | 44 | 22 | 42 |
| East Wild | 39 | 1 | 0 | 0 | 1 | 0 |
| West_Wild | 16 | 0.125 | 0.875 | 0.75 | 0 | 0.25 |
| Total | 55 | 0.745 | 0.255 | 0.218 | 0.709 | 0.073 |
|  |  |  |  |  |  |  |
| SNP3650_6 |  | freq: 2 | freq: 4 | 44 | 22 | 42 |
| East Wild | 40 | 1 | 0 | 0 | 1 | 0 |
| West_Wild | 16 | 0.219 | 0.781 | 0.562 | 0 | 0.438 |
| Total | 56 | 0.777 | 0.223 | 0.161 | 0.714 | 0.125 |
|  |  |  |  |  |  |  |
| SNP2343_45 |  | freq: 1 | freq: 3 | 33 | 11 | 31 |
| East Wild | 42 | 1 | 0 | 0 | 1 | 0 |
| West_Wild | 16 | 0.25 | 0.75 | 0.562 | 0.062 | 0.375 |
| Total | 58 | 0.793 | 0.207 | 0.155 | 0.741 | 0.103 |
|  |  |  |  |  |  |  |
| SNP501_30 |  | freq: 2 | freq: 3 | 22 | 33 | 23 |
| East Wild | 40 | 0.013 | 0.988 | 0 | 0.975 | 0.025 |
| West_Wild | 16 | 0.781 | 0.219 | 0.625 | 0.062 | 0.312 |
| Total | 56 | 0.232 | 0.768 | 0.179 | 0.714 | 0.107 |
|  |  |  |  |  |  |  |
| SNP1284_111 |  | freq: 2 | freq: 3 | 33 | 22 | 32 |
| East Wild | 41 | 0.963 | 0.037 | 0 | 0.927 | 0.073 |
| West_Wild | 16 | 0.188 | 0.812 | 0.75 | 0.125 | 0.125 |
| Total | 57 | 0.746 | 0.254 | 0.211 | 0.702 | 0.088 |
|  |  |  |  |  |  |  |
| SNP6462_91 |  | freq: 2 | freq: 4 | 44 | 22 | 24 |
| East Wild | 42 | 0.714 | 0.286 | 0.071 | 0.5 | 0.429 |
| West_Wild | 16 | 0 | 1 | 1 | 0 | 0 |
| Total | 58 | 0.517 | 0.483 | 0.328 | 0.362 | 0.31 |
|  |  |  |  |  |  |  |
| SNP1204_116 |  | freq: 3 | freq: 4 | 44 | 33 | 43 |
| East Wild | 40 | 0.787 | 0.212 | 0.075 | 0.65 | 0.275 |
| West_Wild | 16 | 0.031 | 0.969 | 0.938 | 0 | 0.062 |
| Total | 56 | 0.571 | 0.429 | 0.321 | 0.464 | 0.214 |
|  |  |  |  |  |  |  |
| SNP5462_40 |  | freq: 1 | freq: 2 | 11 | 22 | 21 |
| East Wild | 34 | 0.294 | 0.706 | 0.147 | 0.559 | 0.294 |
| West_Wild | 14 | 1 | 0 | 1 | 0 | 0 |
| Total | 48 | 0.5 | 0.5 | 0.396 | 0.396 | 0.208 |
|  |  |  |  |  |  |  |
| SNP4231_43 |  | freq: 1 | freq: 3 | 11 | 33 | 31 |
| East Wild | 37 | 0.108 | 0.892 | 0.054 | 0.838 | 0.108 |
| West_Wild | 12 | 0.875 | 0.125 | 0.75 | 0 | 0.25 |
| Total | 49 | 0.296 | 0.704 | 0.224 | 0.633 | 0.143 |
|  |  |  |  |  |  |  |
| SNP1401_14 |  | freq: 1 | freq: 3 | 11 | 33 | 31 |
| East Wild | 40 | 1 | 0 | 1 | 0 | 0 |
| West_Wild | 16 | 0.312 | 0.688 | 0.188 | 0.562 | 0.25 |
| Total | 56 | 0.804 | 0.196 | 0.768 | 0.161 | 0.071 |
|  |  |  |  |  |  |  |
| SNP7369_110 |  | freq: 1 | freq: 3 | 33 | 11 | 13 |
| East Wild | 38 | 0 | 1 | 1 | 0 | 0 |
| West_Wild | 14 | 0.643 | 0.357 | 0.214 | 0.5 | 0.286 |
| Total | 52 | 0.173 | 0.827 | 0.788 | 0.135 | 0.077 |
|  |  |  |  |  |  |  |
| SNP7300_126 |  | freq: 1 | freq: 3 | 11 | 33 | 13 |
| East Wild | 37 | 0 | 1 | 0 | 1 | 0 |
| West_Wild | 13 | 0.615 | 0.385 | 0.385 | 0.154 | 0.462 |
| Total | 50 | 0.16 | 0.84 | 0.1 | 0.78 | 0.12 |
|  |  |  |  |  |  |  |
| SNP14163_31 |  | freq: 3 | freq: 4 | 33 | 44 | 34 |
| East Wild | 37 | 0 | 1 | 0 | 1 | 0 |
| West_Wild | 14 | 0.607 | 0.393 | 0.357 | 0.143 | 0.5 |
| Total | 51 | 0.167 | 0.833 | 0.098 | 0.765 | 0.137 |

**Figure S1.** A genetic Structure plot of the wild-caught UK individuals using data from 8 microsatellites. Each bar represents an individual bird and the proportion of each colour represents the proportion of different ancestry. The plot shows that all the birds have very similar ancestry. The sampling locations are those shown on the map in Figure 1.

**Figure S2.** A genetic structure plot of the wild-caught Greek and North Macedonia individuals using the data from 8 microsatellites. Each bar represents an individual bird and the proportion of each colour represents the proportion of the different genetic ancestry. Three population clusters were present. The sampling locations are those shown on the map in Figure 1.

**Figure S3.** Genetic Structure plot of all birds sampled within the UK. Each bar represents an individual bird and the proportion of each colour represents the proportion of the two genetic ancestries. There is very little differentiation with only two clusters. The one incorporates the Scottish and Northern Irish captive (C) individuals (blue) while the other, most of the wild-caught individuals, along with the captive Suffolk population (green).

**Literature cited**

Bech, N. *et al.* (2010) ‘Transferability of microsatellite markers among economically and ecologically important galliform birds’, *Genetics and Molecular Research*, 9(2), pp. 1121–1129. doi: 10.4238/vol9-2gmr760.

Ferrero, M. E. *et al.* (2007) ‘Sixteen new polymorphic microsatellite markers isolated for red-legged partridge (Alectoris rufa) and related species’, *Molecular Ecology Notes*, 7(6), pp. 1349–1351. doi: 10.1111/j.1471-8286.2007.01877.x.
